# Growth-Blocking Peptides As Nutrition-Sensitive Signals for Insulin Secretion and Body Size Regulation

**DOI:** 10.1371/journal.pbio.1002392

**Published:** 2016-02-29

**Authors:** Takashi Koyama, Christen K. Mirth

**Affiliations:** 1 Instituto Gulbenkian de Ciência, Oeiras, Portugal; 2 School of Biological Sciences, Monash University, Clayton, Victoria, Australia; MRC National Institute for Medical Research, UNITED KINGDOM

## Abstract

In *Drosophila*, the fat body, functionally equivalent to the mammalian liver and adipocytes, plays a central role in regulating systemic growth in response to nutrition. The fat body senses intracellular amino acids through Target of Rapamycin (TOR) signaling, and produces an unidentified humoral factor(s) to regulate insulin-like peptide (ILP) synthesis and/or secretion in the insulin-producing cells. Here, we find that two peptides, Growth-Blocking Peptide (GBP1) and CG11395 (GBP2), are produced in the fat body in response to amino acids and TOR signaling. Reducing the expression of GBP1 and GBP2 (GBPs) specifically in the fat body results in smaller body size due to reduced growth rate. In addition, we found that GBPs stimulate ILP secretion from the insulin-producing cells, either directly or indirectly, thereby increasing insulin and insulin-like growth factor signaling activity throughout the body. Our findings fill an important gap in our understanding of how the fat body transmits nutritional information to the insulin producing cells to control body size.

## Abstract 2

The insect fat body transmits nutritional information to control body growth by producing and secreting two peptides, GBP1 and GBP2, in response to dietary amino acids. These two peptides stimulate insulin-like peptide secretion, increasing insulin-signaling activity throughout the body and inducing systemic growth.

## Author Summary

Organisms adjust their development in response to environmental conditions to maximize important life history traits such as body size and survival. From work in the fruit fly, *Drosophila melanogaster*, we are beginning to resolve some of the molecular mechanisms through which environmental conditions, specifically nutrition, modify developmental processes. The insect fat body is functionally equivalent to the mammalian liver and adipocytes. In response to the concentration of dietary amino acids, the fat body secretes a peptide signal into the insect bloodstream that regulates the systemic release of important growth-regulating peptides, the insulin-like peptides. The nature of this peptide signal was previously unknown. Here we found that two peptides, Growth-Blocking Peptide (GBP1) and CG11395 (GBP2), are produced in the fat body in response to amino acids. Once secreted from the fat body, these two peptides stimulate secretion of insulin-like peptides, which results in elevating insulin-signaling activity in the rest of the body to stimulate body growth.

## Introduction

The nutritional environment of developing animals can generate dramatic variation in body size and shape, as well as in their developmental time. Incorrect regulation of body size has important consequences on the adult animal, impacting its survival and its fitness by altering its fecundity, longevity, and stress resistance [1–4]. For this reason, animals keep their growth rates and the duration of their growth period tightly in tune with nutritional availability. How nutritional conditions are sensed and relayed to the body’s organs to control growth and development remains not well understood.

Over the past 15 y, work in the fruit fly, *Drosophila melanogaster*, has provided significant insight into how nutrition regulates the growth of the body [1,5–9]. In insects, final body size is mostly a product of growth in the larval stages. In larvae, the fat body plays a central role in regulating systemic growth in response to dietary protein [10]. The fat body senses the concentration of intracellular amino acids through the activity of the Target of Rapamycin (TOR) signaling pathway, which in turn regulates the synthesis and secretion of an undescribed humoral factor(s), called the fat-body–derived signal. This signal regulates systemic growth by controlling the synthesis and secretion of insulin-like peptides (ILPs) by the insulin-producing cells of the brain [10].

Once secreted into the bloodstream, ILPs act on the organs of the body to regulate their growth, thereby regulating body size in accordance with nutrition [3,11]. Decreasing insulin and insulin-like growth factor signaling (IIS) throughout the entire body leads to decreased growth rates and increased development time [12], similar to the phenotypes induced by starvation [13]. Because the insulin-producing cells do not directly sense amino acid concentrations, understanding the nature of the humoral factor linking amino acid-sensing in the fat body to ILP secretion in the brain is central to our understanding of how nutrition regulates developmental processes.

Despite its importance, the nature of the fat-body–derived signal has remained unknown. Based on a number of studies, we postulate that to transmit nutritional information from the fat body to the insulin-producing cells, the fat-body–derived signal would need to have the following properties: (1) it should be produced in the fat body and secreted into the hemolymph, (2) its expression and/or secretion should be amino acid- and TOR-sensitive, (3) it should act downstream of TOR signaling to stimulate ILP secretion from the insulin-producing cells, and (4) its secretion should increase IIS activity in the entire body, resulting in increased body size.

The fat body secretes several molecules that could potentially act as fat-body–derived signals. Recent studies identified sugar- and lipid-sensitive humoral signals secreted by the fat body, Unpaired 2 and CCHamide-2 [14,15]. Although Unpaired 2 and CCHamide-2 stimulate ILP secretion from the insulin-producing cells, their secretions are not sensitive to amino acids. Since larval growth relies primarily on protein, the fat-body–derived signal is unlikely to be Unpaired 2 or CCHamide-2 [10].

The larval fat body also secretes an epidermal growth factor-like peptide known as Growth-Blocking Peptide (GBP). GBP was originally described in a lepidopteran, the armyworm, *Pseudaletia separata*, and regulates growth and immune response in a concentration-dependent manner [16,17]. In *Drosophila*, the GBP paralog (GBP1 in this study) and two GBP homologs, CG11395 (GBP2) and CG17244 (GBP3), are expressed in the larval fat body [18]. GBP1 regulates immune response in *Drosophila*. Furthermore, GBP1 and GBP2 are secreted into the hemolymph in *Drosophila* [18,19] and GBP1 expression has been shown to be sensitive to starvation and TOR signaling [20,21]. Thus, we hypothesized that GBPs may act as fat-body–derived signals.

In this study, we provide evidence that GBP1 and GBP2 bear the properties we propose for the fat-body–derived signal. Both GBP1 and GBP2 are synthesized in the fat body in response to amino acids and TOR signaling. Modifying their expression in the fat body affects ILP secretion from the insulin-producing cells, thereby changing ILP concentrations in the circulating hemolymph. This results in changing IIS activity throughout the entire body, thereby regulating body size. Our data therefore fill an important gap in our understanding of how nutrition regulates body size, by uncovering the molecular mechanisms through which the fat body communicates nutritional information to regulate ILP synthesis and secretion.

## Results

### Modifying TOR Signaling in the Fat Body Alters Body Size

**Fig 1:**
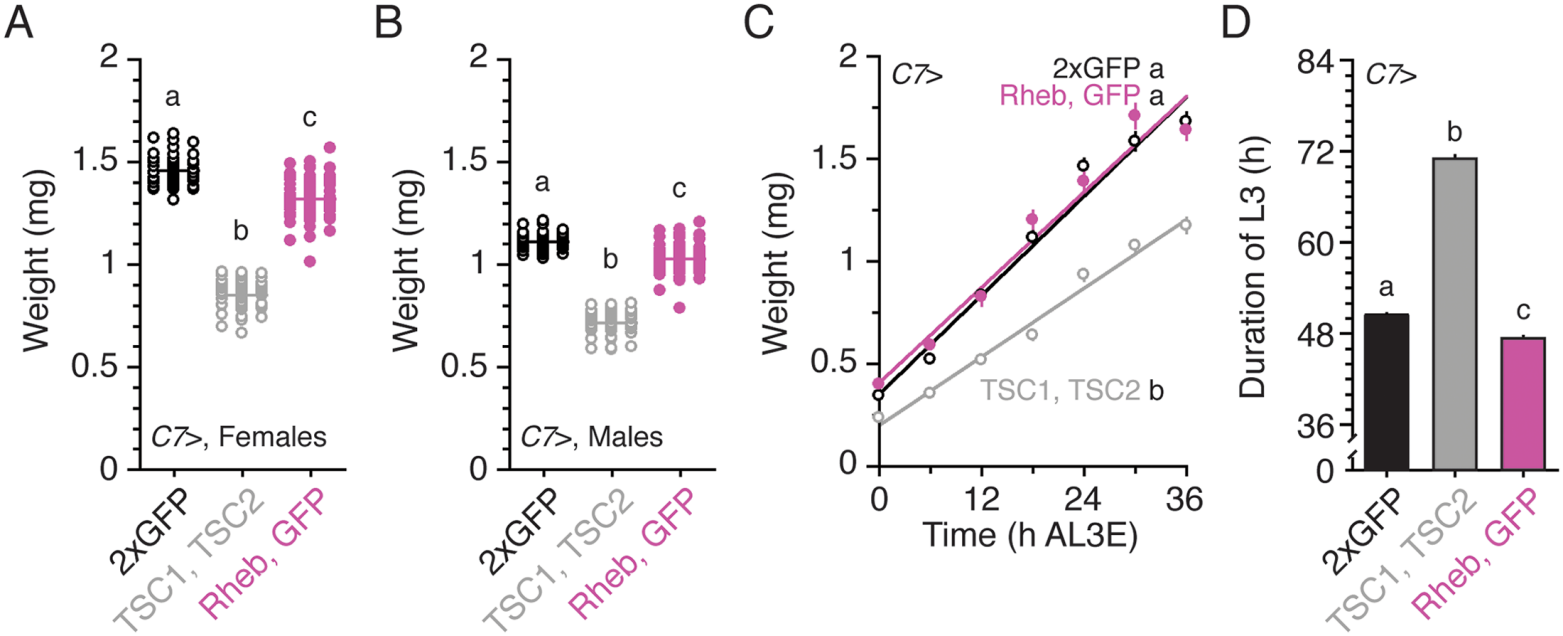
Changing TOR signaling activity in the fat body affects body size. **(A, B)** Changing TOR signaling in the fat body affects final body size in females (A) and males (B). *n* = 47–72 for A and *n* = 44–70 for B. Pharate adults (approximately 2–14 h before eclosion) were weighed as a proxy of final adult size. **(C)** Activating TOR signaling in the fat body does not affect growth rate while repressing TOR signaling reduces growth rate. *n* = 14–17/time point. **(D)** Activating TOR signaling in the fat body slightly shortens the duration of the L3. Repressing TOR signaling in the fat body extends the duration of the L3. *n* = 77–113. Two copies of UAS transgenes were expressed using the *C7* Gal4 driver in all experiments. Treatments sharing the same letter indicate the groups that are statistically indistinguishable from one another (*p* < 0.05, ANOVA and pairwise *t* tests). Growth rate was analyzed by ANCOVA and post hoc comparisons of the slopes. The supplementary file in which the data used to generate each plot can be found is S1 Data.

Previous studies have shown that changing the levels of TOR signaling in the larval fat body results in the regulation of ILP secretion [10]. To address whether GBPs are humoral signals that convey the level of TOR signaling in the fat body to the insulin-producing cells, we first altered TOR signaling in the fat body and assessed the effects on adult body size, as determined by weighing pharate adults immediately prior to eclosion. To repress TOR signaling in the fat body, we co-overexpressed TSC1 and TSC2, proteins that form a complex to down-regulate TOR activity [22,23], specifically in this tissue using a fat-body specific *C7* Gal4 driver [24]. Using the G-TRACE method [25], which allows us to identify both lineage-traced expression (marked with green fluorescent protein [GFP]) and live Gal4 (marked with DsRed), we found that *C7* Gal4 expressed Gal4 strongly in the fat body and salivary glands at 24 h after L3 ecdysis (AL3E) (S1A Fig). Furthermore, this line expressed Gal4 in a small region of the wing pouch and a number of neurons during the younger developmental stages. Similar to previous reports [10,14,26], reducing TOR activity by overexpressing both TSC1 and TSC2 in the fat body decreased body size in both females and males (Fig 1A and 1B). These effects on body size were due to a decrease in larval growth rates (Fig 1C) and were accompanied by an increase in larval developmental time (Fig 1D), similar to the effects seen when IIS signaling is suppressed throughout the animal or when animals are starved [12]. Therefore, these results suggest that reducing TOR signaling activity in the fat body genocopies the effect of starvation. Interestingly, we found that hyper-activating TOR signaling by overexpressing Rheb in the fat body induced mild reductions in body size (Fig 1A and 1B) by shortening the duration of the third larval instar (L3) without affecting growth rate (Fig 1C and 1D). Our results confirm that the levels of TOR signaling in the fat body regulate adult body size.

### Reducing GBP1 and GBP2 Expression Reduces Body Size

**Fig 2:**
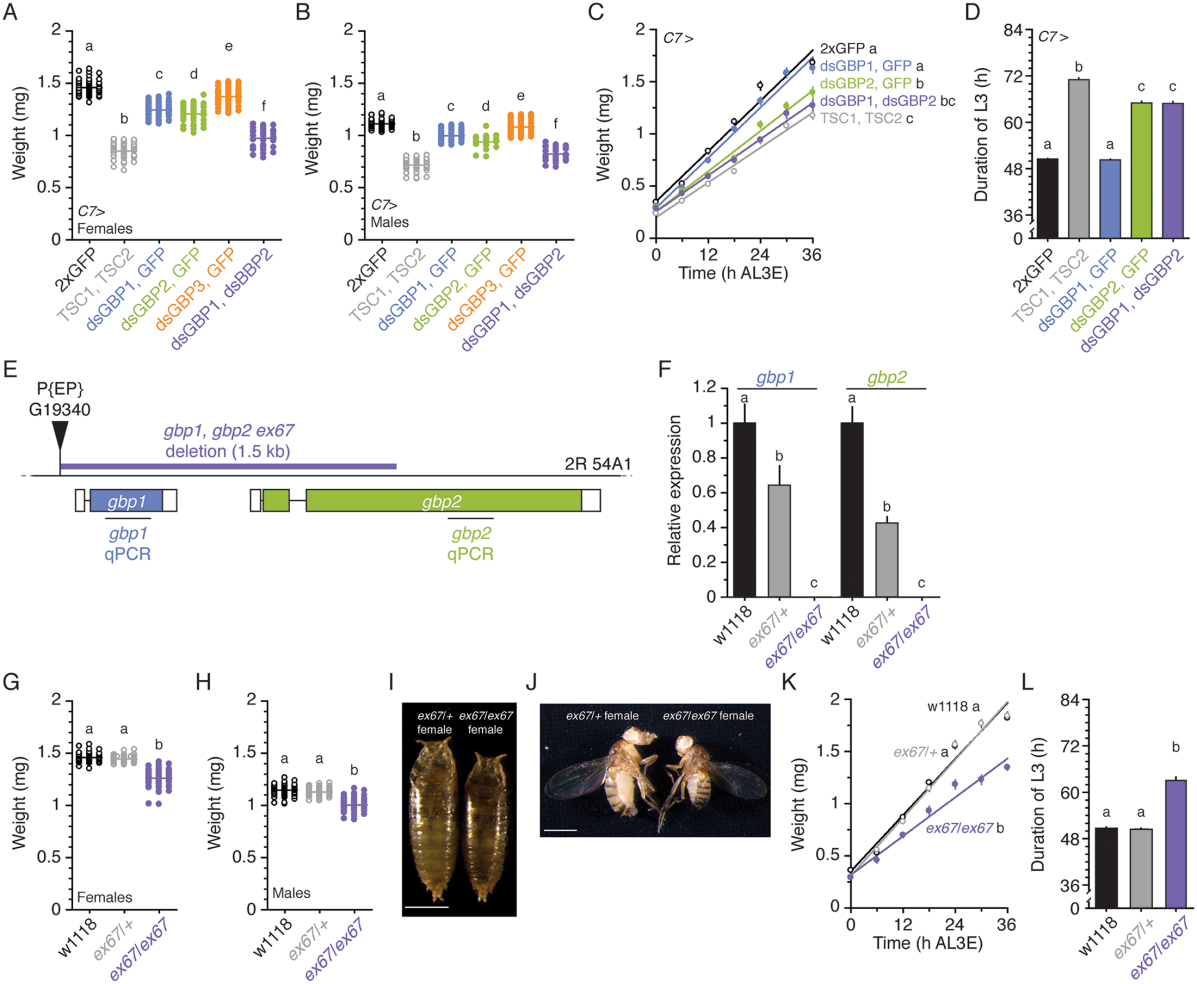
Reducing GBP1 and GBP2 expression in the fat body reduces body size due to reduced growth rate. **(A, B)** GBP1, GBP2 double-knockdown in the fat body reduces final body size in females (A) and males (B). *n* = 35–65 for A and *n* = 21–56 for B. **(C)** GBP1, GBP2 double-knockdown in the fat body reduces growth rate. *n* = 14–17/time point. **(D)** GBP1, GBP2 double-knockdown extends the duration of the L3. *n* = 77–115. To compensate the effect of transgenes, we overexpressed two copies of UAS transgenes using the *C7* Gal4 driver for A–D. **(E)** *gbp1*, *gbp2 ex67* mutation was created using a P-element excision mutagenesis method. The *gbp1*, *gbp2 ex67* mutant lacks 1525 bp from the P-element insertion site, which includes the entire *gbp1* gene and one-third of the second exon of *gbp2* gene. Open boxes indicate untranslated regions and colored boxes indicate open reading frames. *gbp1* qPCR and *gbp2* qPCR indicate the region we amplified for the evaluation of mRNA expression in the *gbp1*, *gbp2 ex67* mutant by qPCR in F. **(F)** The *gbp1*, *gbp2 ex67* mutant shows no *gbp1* and *gbp2* mRNA expression. We normalized the values using an internal control, *RpL3*. Then, we standardized the expression level of each gene by fixing the values from w1118 animals to 1. We used five larvae for each sample and five biologically independent samples for each genotype. Each bar indicates the relative mean expression ± standard error of the mean (SEM). **(G, H)** *gbp1*, *gbp2 ex67* mutant animals show reduced body size both in females (G) and males (H). *n* = 49–69 for G and *n* = 49–76 for H. **(I, J)** *gbp1*, *gbp2 ex67* mutant animals are smaller than heterozygous animals. Pupae shortly before eclosion (I) and 1-d-old female adults (J) of mutant and heterozygote animals were photographed. Scale bars are 1 mm. **(K)** *gbp1*, *gbp2 ex67* mutant shows reduced growth rate. *n* = 14–43/time point. **(L)** *gbp1*, *gbp2 ex67* mutant shows extended duration of the L3. *n* = 84–109. Treatments sharing the same letter indicate the groups that are statistically indistinguishable from one another (*p* < 0.05, ANOVA and pairwise *t* tests). Growth rate was analyzed by ANCOVA and post hoc comparisons of the slopes. The supplementary file in which the data used to generate each plot can be found is S1 Data.

Next, we aimed to test our hypothesis that GBPs act as fat-body–derived signals. To do this, we first knocked down three GBP-like genes expressed in the larval fat bodies, GBP1, GBP2, and GBP3, specifically in the fat body and assessed the effects on final body size. When we knocked down either GBP1 or GBP2 using a *C7* Gal4 driver, both females and males showed reduced body size (Fig 2A and 2B). Knocking down GBP3 induced more subtle, albeit significant, size reductions. Since all three RNAi constructs were almost equally effective at reducing the levels of mRNA transcripts in our conditions (S2 Fig), and because GBP1 and GBP2 appeared to account for greater effects on body size, we excluded GBP3 for further analyses. We then knocked down GBP1 and GBP2 simultaneously in the larval fat body and found that these animals were two-thirds the size of the control animals (Fig 2A and 2B).

We next determined whether this size reduction was due to reduced growth rate or to alterations in developmental timing. Compared to control larvae, knocking down GBP2 in the fat body induced slower growth (Fig 2C). Knocking down GBP1 did not change growth rate. Simultaneous knockdown of GBP1 and GBP2 showed more severe growth retardation, and this reduction in growth rate was indistinguishable from that induced by overexpressing TSC1 and TSC2 in the fat body.

Finally, we measured the duration of the L3 as a proxy of the duration of the feeding period in those animals, since when growth rate is reduced due to reduced IIS activity, larvae also extend the duration of the feeding period [12,27,28]. We found that when we knocked down GBP2 in the fat body, larvae extended their feeding period. We saw no changes in developmental timing when we knocked down GBP1 (Fig 2D). Although in the fat body of larvae staged 24 h AL3E, *gbp1* expression is stronger than that of *gbp2* (S3 Fig), both GBP1 and GBP2 play roles in regulating growth.

To confirm that the reductions in body size were due to reduced GBP1 and GBP2 expression in the fat body, we quantified mRNA expression of *gal4*, *gbp1*, and *gbp2* in various organs at the 24 h AL3E by qPCR in *C7* Gal4 larvae. We detected both *gbp1* and *gbp2* mRNAs only in the fat bodies besides weak *gbp1* expression in the malpighian tubles (S1B Fig). *Gal4* mRNA was expressed strongly in the fat body and salivary glands, and basal *gal4* mRNA was detected in various organs (S1B Fig). Since expression patterns of *gal4*, and *gbp1* or *gbp2* only overlap in the fat body, these observations suggest that the growth effects we observed are mainly derived from the fat body.

As an additional validation, we used a second fat-body expressed Gal4 driver, *pumpless* Gal4 (*ppl* Gal4) [29], to knock down GBP1 and/or GBP2 to validate the phenotypes observed using the *C7* Gal4 (S4 Fig). When we knocked down GBP1 and GBP2, either alone or in combination, using *ppl* Gal4, the resulting animals showed reduced body size, reduced growth rate, and extended growth duration. Taken together, we concluded that GBP1 and GBP2 regulate size and growth mainly via their secretion from the fat body.

Finally, we further validated the effects of GBP1 and GBP2 on size regulation by generating a mutant for both *gbp1* and *gbp2*, which are positioned next each other on the second chromosome. We used the P-element insertion line, P{EP} G19340, whose insertion locus is 80 bp upstream of the 5′-end of the open reading frame of the *gbp1* gene (Fig 2E). By performing a P-element excision, we deleted 1525 bp of this region, disrupting both *gbp1* and *gbp2* (*gbp1*, *gbp2 ex67*). This deletion removes the entire *gbp1* open reading frame and 594 bp of 5′-end of *gbp2* open reading frame. In mutant larvae, we could not detect either *gbp1* or *gbp2* mRNA using quantitative PCR (qPCR) (Fig 2F). Thus, we concluded that the *gbp1*, *gbp2 ex67* mutant is null for both genes.

Using this line, we explored whether the *gbp1*, *gbp2 ex67* mutant shows phenotypes similar to GBP1, GBP2 double knockdown animals. As we expected, these animals showed reduced body size (Fig 2G–2J), slower growth rate (Fig 2K), and extended duration of the L3 (Fig 2L). Interestingly, although heterozygous mutant larvae showed reduced expression of both *gbp1* and *gbp2*, the body size, growth rate, and the duration of the L3 were indistinguishable from wild-type larvae. This might be because moderate reduction in the expression of these two genes was not sufficient to induce alterations in either phenotype. Taken together, we concluded that GBP1 and GBP2 regulate size by controlling growth rate.

### Amino Acids Regulate *gbp1* and *gbp2* Expression in the Fat Body via TOR Signaling

Our results show that GBP1 and GBP2 regulate body size, and that interfering with their activity genocopies the effects of starvation: reducing growth rates and extending the duration of the L3. However, to transmit nutritional information from the fat body to the insulin-producing cells, we proposed that the fat-body–derived signal would need to be sensitive to both the amino acid concentration of the larval food and to TOR signaling in the fat body. Thus, we next examined whether mRNA expression of *gbp1* and *gbp2* is sensitive to amino acids and to TOR signaling.

**Fig 3:**
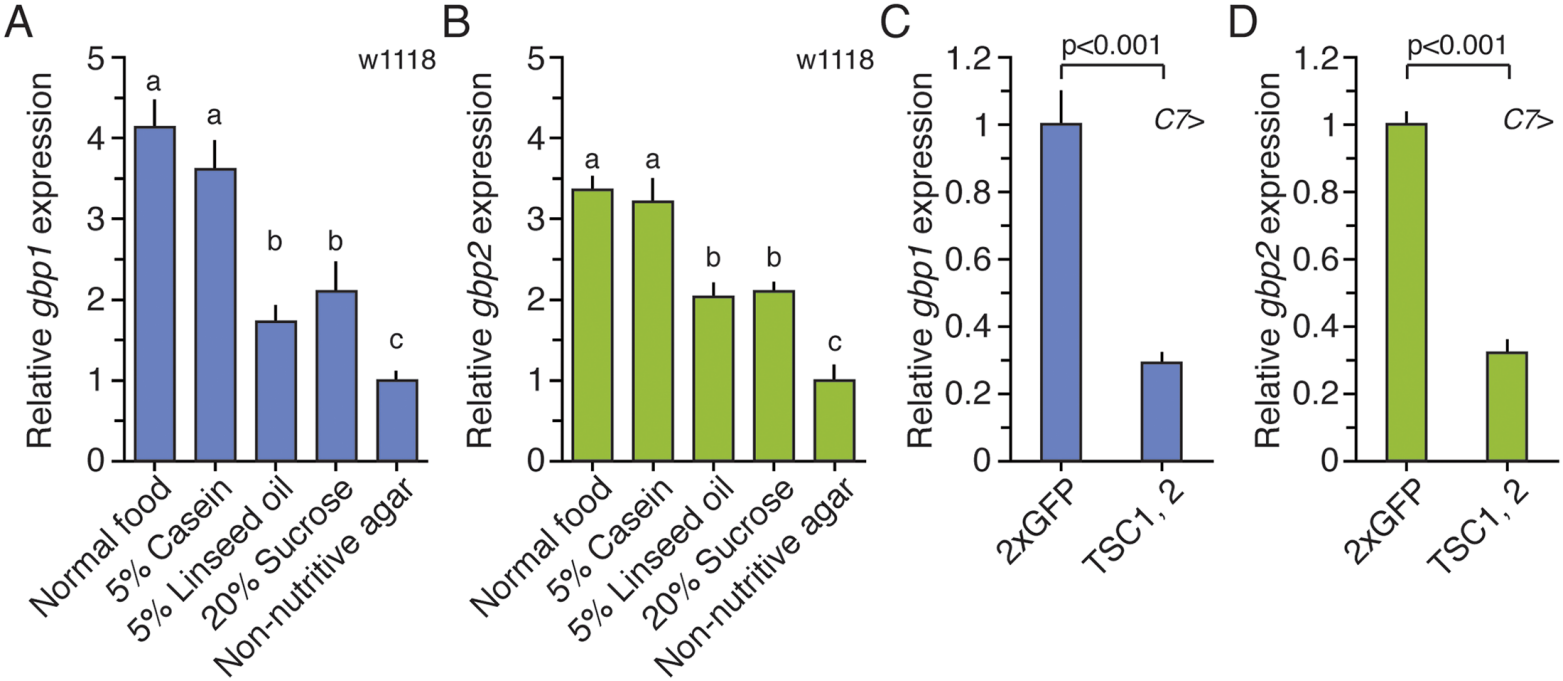
Nutrition regulates *gbp1* and *gbp2* expression via TOR signaling in the fat body. **(A, B)** Amino acid intake is sufficient to induce *gbp1* (A) and *gbp2* (B) mRNA expression in the fat body of w1118 larvae. Larvae were staged at the onset of the L3, and fed on normal food for 12 h. Then they were starved for 12 h on 1% non-nutritive agar followed by an additional 12 h on one of five nutritionally different media. Columns sharing the same letter indicate the groups that are statistically indistinguishable from one another (ANOVA and pairwise *t* tests, *p* < 0.05). **(C, D)** Reducing TOR signaling activity in the fat body decreases *gbp1* (C) and *gbp2* (D) mRNA expression in the fat body. Larvae were staged at the onset of the L3, and fed on normal food for 24 h. Numbers indicate p-values (ANOVA and pairwise *t* tests). We normalized the values using an internal control, *RpL3*. Then, we standardized the expression level of each gene by fixing the values from non-nutritive agar treated animals to 1 in A and B, and from *C7*>2xGFP to 1 in C and D. We used the fat bodies from five larvae for each sample and five biologically independent samples for each condition. Each bar indicates the relative mean expression ± SEM. The supplementary file in which the data used to generate each plot can be found is S1 Data.

We carefully staged wild-type (w1118) larvae at the molt to the L3, fed them for 12 h, and then starved them for 12 h on non-nutritive agar to eliminate the effect of normal fly food. Then, we re-fed these larvae on one of five different foods: normal food, 5% casein (amino acid source), 5% linseed oil (lipid source), 20% sucrose (sugar source), or non-nutritive agar for an additional 12 h. We then dissected the fat bodies from these larvae, and used qPCR to quantify the effects of larval diet composition on *gbp1* and *gbp2* expression. We found that expression levels of both *gbp1* and *gbp2* were significantly reduced in larvae fed on agar when compared to larvae fed on normal food (Fig 3A and 3B). Feeding larvae on casein alone was sufficient to restore *gbp1* and *gbp2* mRNA levels to those of larvae fed on standard food. Interestingly, although larvae fed on either linseed oil or sucrose showed significantly higher expression of *gbp1* and *gbp2* when compared to those fed on agar, the effects on up-regulating *gbp1* and *gbp2* expression were significantly lower than that of casein. Finally, when we overexpressed TSC1 and TSC2 in the fat body to reduce TOR activity, both *gbp1* and *gbp2* expression was significantly reduced in the fat body (Fig 3C and 3D). Thus, the mRNA expression of *gbp1* and *gbp2* is sensitive to amino acids, lipids, sugar, and TOR signaling.

### GBP1 and GBP2 Stimulate ILP2 and ILP5 Secretion from the Insulin-Producing Cells

The phenotypes we observed in double knock down and double null mutant animals are typical of those observed in ILP-deficient animals and in animals with reduced IIS [3, 11,30]. Furthermore, we determined that the expression of both *gbp1* and *gbp2* respond to both amino acid concentration in the diet and TOR signaling. To test whether GBP1 and GBP2 transmit nutritional information from the fat body to the insulin-producing cells, we determined whether GBP1 and GBP2 control ILP secretion.

**Fig 4:**
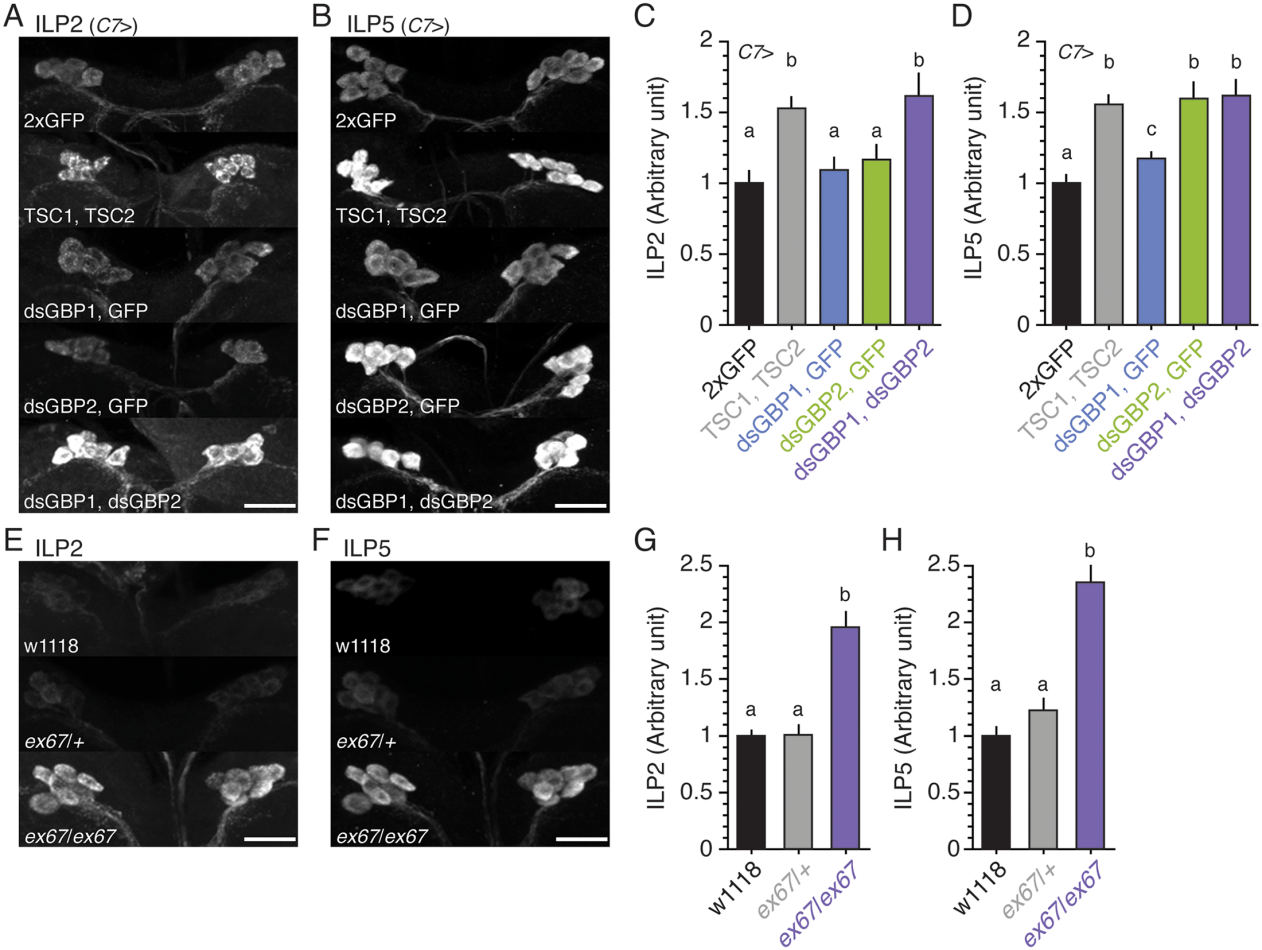
GBP1 and GBP2 regulate ILP2 and ILP5 secretion. **(A, B)** Knocking down GBP1 and GBP2 increases ILP2 (A) and ILP5 (B) accumulation in the insulin-producing cells. The scale bars are 20 μm. **(C, D)** Knocking down GBP1 and GBP2 increases the densities of ILP2 (C) and ILP5 (D) signals in the insulin-producing cells. We standardized the densities of ILPs by fixing the values from *C7*>2xGFP to 1. *n* = 16–27 for both ILP2 and ILP5. **(E, F)** The *gbp1*, *gbp2 ex67* mutant shows increased ILP2 (E) and ILP5 (F) accumulation in the insulin-producing cells. The scale bars are 20 μm. **(G, H)** The *gbp1*, *gbp2 ex67* mutant shows increased densities of ILP2 (G) and ILP5 (H) signals in the insulin-producing cells. We standardized the densities of ILPs by fixing the values from w1118 larvae to 1. *n* = 34–41 for both ILP2 and ILP5. Treatments sharing the same letter indicate the groups that are statistically indistinguishable from one another (ANOVA and pairwise *t* tests, *p* < 0.05). The supplementary file in which the data used to generate each plot can be found is S1 Data.

To examine whether GBP1 and GBP2 control ILP secretion, we immunostained larval central nervous systems for both ILP2 and ILP5. When TOR signaling is reduced in the fat body, the insulin-producing cells do not secrete ILPs. This results in ILP accumulation in these cells, a feature that is commonly used as a proxy for levels of circulating ILPs [10,14,15,31–33]. In the control condition, we detected moderate accumulation of ILP2 and ILP5 in the insulin-producing cells (Fig 4A and 4B). In contrast, when we reduced TOR signaling by overexpressing TSC1 and TSC2 in the fat body using *C7* Gal4, we observed strong accumulation of ILP2 and ILP5 in these cells. Knocking down either GBP1 or GBP2 significantly increased the accumulation of ILP5, although ILP2 accumulation was not affected (Fig 4A–4D). When we knocked down GBP1 and GBP2 simultaneously in the fat body, the level of both ILP2 and ILP5 accumulation increased, in a manner similar to when TSC1 and TSC2 were overexpressed in this tissue (Fig 4A–4D). Similar accumulation of ILPs was observed when we knocked down GBP1 and/or GBP2 using the *ppl* Gal4 (S5 Fig).

We also found that both ILP2 and ILP5 accumulated in the insulin-producing cells of *gbp1*, *gbp2* null mutant larvae when compared to wild-type or heterozygous mutant animals (Fig 4E–4H). These results indicate an involvement of GBP1 and GBP2 in ILP secretion from the insulin-producing cells, which would result in a decrease of IIS activity in the entire body.

Since *ilp5* mRNA expression is known to be sensitive to nutrition, we also quantified *ilp2* and *ilp5* mRNA when both GBP1 and GBP2 were knocked down in the fat body and in *gbp1*, *gbp2 ex67* mutant larvae. As expected, *ilp5* mRNA expression was lower both when GBP1, GBP2 were both knocked down in the fat body and in the *gbp1*, *gbp2 ex67* mutant larvae (S6B and S6D Fig). On the other hand, in larvae where TOR activity is reduced and both GBP1 and GBP2 are knocked down, we found that *ilp2* mRNA expression was elevated (S6A Fig). We did not find this effect in *gbp1*, *gbp2 ex67* mutant larvae (S6C Fig). This difference in the effect on *ilp2* mRNA expression could result from the difference between completely removing the activity of these genes in the *gbp1*, *gbp2 ex67* mutant larvae versus partial reduction of their mRNAs in the GBP1, GBP2 double knock downs. Alternatively, it could be because GBP1 and GBP2 function in other organs to regulate *ilp2* expression.

**Fig 5:**
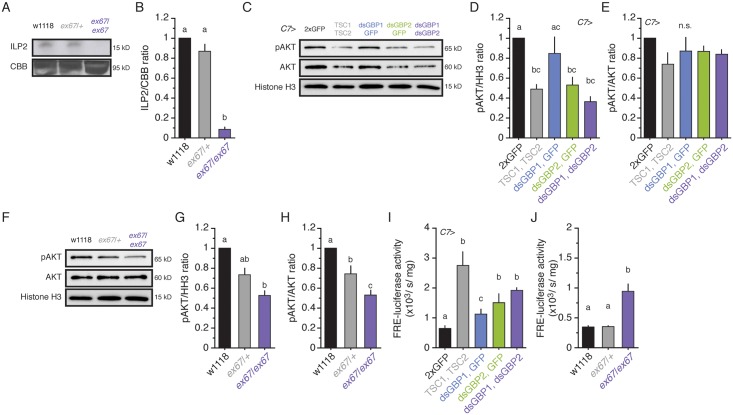
GBP1 and GBP2 regulate IIS activity in the entire body. **(A)** Concentration of circulating ILP2 is reduced in the *gbp1*, *gbp2 ex67* mutant. After SDS-PAGE, the gel was cut and upper half was stained with Coomassie Brilliant Blue (CBB) and putative larval serum protein 1α, β and γ proteins (90–100 kDa) were shown as a loading control. The lower half was used for ILP2 staining. (**B**) ILP2 signal density was quantified from three biologically independent experiments against putative larval serum protein 1α, β and γ proteins. We standardized the densities of all three proteins by fixing the values from w1118 to 1. **(C)** Knocking down GBP1 and GBP2 in the fat body shows reduced pAKT in the wing discs. AKT and Histone H3 were used as loading controls. (**D, E**) pAKT signal density was quantified from three biologically independent experiments against Histone H3 (D) and AKT (E). We standardized the densities of all three proteins by fixing the values from *C7*>2xGFP to 1. **(F)** The *gbp1*, *gbp2 ex67* mutant shows reduced pAKT in the wing discs. AKT and Histone H3 were used as loading controls. (**G, H**) pAKT signal density was quantified from three biologically independent experiments against Histone H3 (G) and AKT (H). We standardized the densities of all three proteins by fixing the values from w1118 to 1. **(I)** Knocking down GBP1 and GBP2 increases FRE-luciferase activity in the entire body. Larvae were staged at the onset of the L3, and fed on normal food for 24 h. For the luciferase assay, we used the entire bodies of five larvae. We quantified five replicates for each genotype. **(J)** The *gbp1*, *gbp2 ex67* mutant shows increased FRE-luciferase activity in the entire body. We quantified five replicates for each genotype. Treatments sharing the same letter indicate the groups that are statistically indistinguishable from one another (ANOVA and pairwise *t* tests, *p* < 0.05). The supplementary file in which the data used to generate each plot can be found is S1 Data.

To confirm whether ILP accumulation in the insulin-producing cells actually results in a decrease of ILP2 concentration in the circulating hemolymph, we collected hemolymph from wild-type, and *gbp1*, *gbp2 ex67* heterozygous and homozygous mutant larvae at 24 h AL3E, and performed western blot analyses using an anti-ILP2 antibody. In the homozygous null mutant, we could not detect circulating ILP2 although we detected it in the hemolymph of wild-type and heterozygous animals (Fig 5A and 5B). This not only shows that GBP1 and GBP2 regulate ILP2 secretion, it further validates the method of using ILP2 and ILP5 accumulation in the insulin-producing cells as a proxy for ILP secretion.

Reductions in circulating ILPs are predicted to reduce IIS activity throughout the body. By binding to the insulin receptor, ILPs induce a phosphorylation cascade that ultimately induces growth in the target tissues [1,5,7]. For example, ILPs induce the phosphorylation of AKT, which in turn phosphorylates the negative growth regulator Forkhead Box O (FoxO). FoxO is a transcription factor, and its phosphorylation causes it to be moved out of the nucleus preventing it from binding to its target genes. Measuring the levels of AKT phosphorylation and FoxO transcriptional activity provide two useful readouts of IIS levels.

We first assessed systemic levels of IIS signaling by performing western blot analyses to measure phospho-AKT levels in the wing discs. As we expected, phospho-AKT level was reduced in wing discs from larvae in which both GBP1 and GBP2 were knocked down in the fat body (Fig 5C–5E) and in wing discs from the *gbp1*, *gbp2* null mutant larvae (Fig 5F–5H). Interestingly, when we knocked down these two genes or reduced TOR activity specifically in the fat body, both total AKT and pAKT levels were reduced. We did not observe a reduction in total AKT in the double mutant. Again, this might be either due to the difference between complete knock out of the genes versus reduction of *gbp1*, *gbp2* expression via RNAi, or due to the activity of GBP1 and GBP2 in other organs.

Next, we used the FoxO-Response Element (FRE)-luciferase construct to obtain a quantitative measure of FoxO activity [34–36]. Since FoxO is a negative regulator of IIS, high luciferase activity indicates low IIS activity in the entire body. We found that FRE activity was significantly higher when we reduced TOR activity or knocked down GBP1 and/or GBP2 in the fat body when compared to control larvae (Fig 5I). Similarly, FRE activity was higher in *gbp1*, *gbp2* double mutant animals than it was in heterozygous or w1118 larvae (Fig 5J). These results further support the notion that nutrition-sensitive expression of GBP1 and GBP2 controls IIS activity throughout the body.

### Overexpressing GBP1 and GBP2 Rescues the Effects of the *gbp1*, *gbp2* Null Mutant on Body Size

To analyze the function of GBP1 or GBP2 more precisely, we created UAS-mediated *gbp1* and *gbp2* overexpression constructs using the phiC31 integrase and an attP2 landing site-carrying recipient line [37]. Since it is known that GBP regulates growth in a concentration-dependent manner in lepidopterans [16], we used the temperature-sensitive properties of Gal4 to modify the level of transgene expressed in the wild-type background by rearing larvae either at 22 or 25°C. We found that although both GBP1 and GBP2 overexpression induced slight increases in body size at 25°C, these transgenes induced greater increases in body size at 22°C for both females and males (S7A–S7D Fig). In lepidopterans, GBP induces growth in a dose-dependent manner, however high concentrations suppress growth [16,17]. Indeed, we found that both genes were expressed at higher levels at 25°C than at 22°C (S7E and S7F Fig). We could not detect significant differences in ILP2 and ILP5 accumulation between 22 and 25°C (S7G–S7J Fig). Nevertheless, the more subtle effects on body size that we observe in *Drosophila* body size at 25°C may be because the level of GBP expressed is within the range where it begins to suppress growth, even if the effects in insulin secretion are below our level of detection. For these reasons, we performed all GBP overexpression experiments at 22°C.

**Fig 6:**
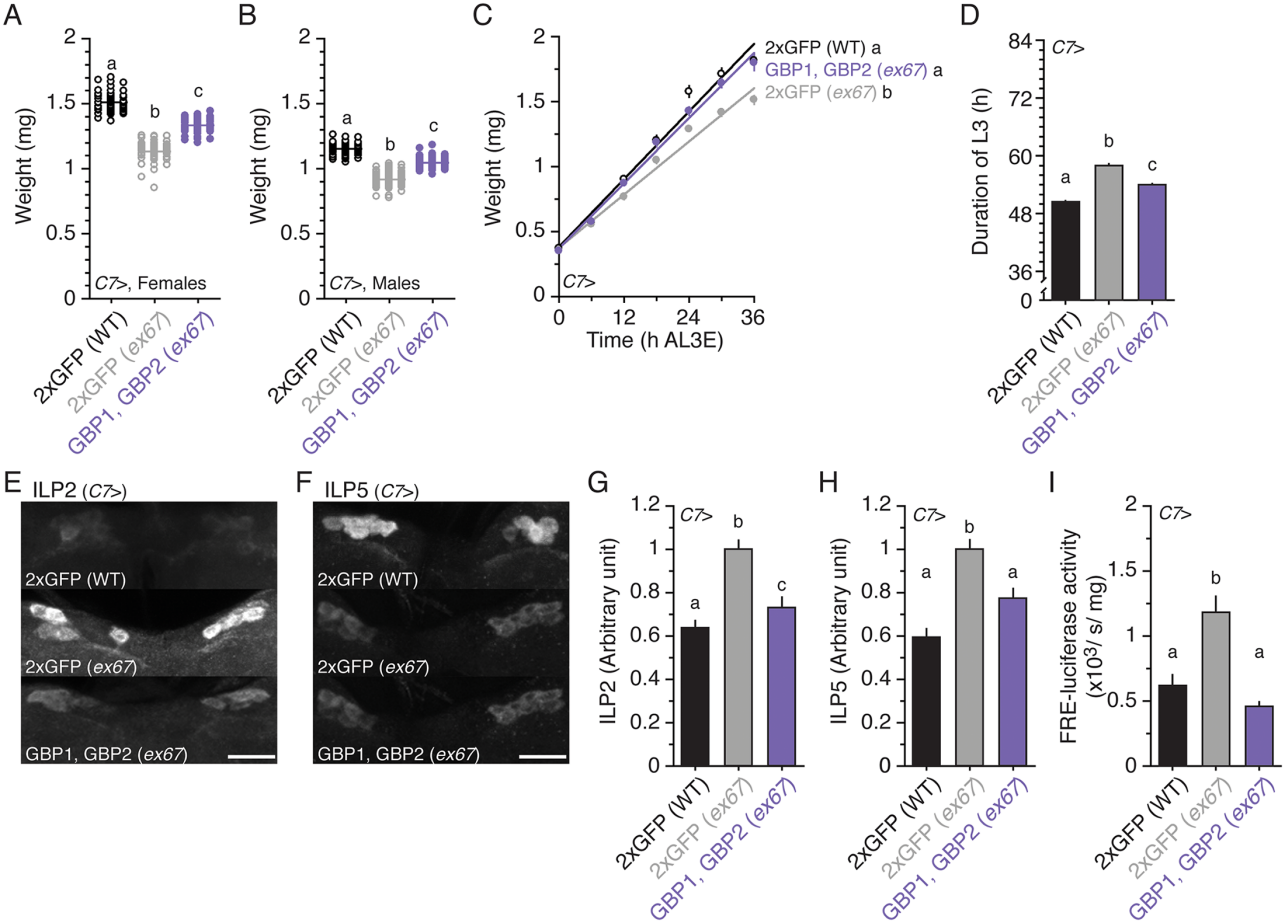
Overexpressing GBP1 and GBP2 in the fat body rescues body size in *gbp1*, *gbp2 ex67* null mutant larvae. **(A, B)** Overexpressing both GBP1 and GBP2 partially rescues body size reduction in *gbp1*, *gbp2 ex67* mutant females (A) and males (B) at 22°C. *n* = 51–52 for A and *n* = 54–65 for B. **(C)** Overexpressing both GBP1 and GBP2 partially increases growth rate in *gbp1*, *gbp2 ex67* mutant larvae. *n* = 14–20/time point. **(D)** Overexpressing both GBP1 and GBP2 partially rescues the duration of the L3 in *gbp1*, *gbp2 ex67* mutant larvae. *n* = 111–119. **(E, F)** Overexpressing both GBP1 and GBP2 reduces ILP2 (E) and ILP5 (F) accumulation in the insulin-producing cells of *gbp1*, *gbp2 ex67* mutant larvae. Larvae were staged at the onset of the L3, and then fed on normal food for 24 h. The insulin-producing cells were immunostained using an anti-ILP2 antibody and an anti-ILP5 antibody. **(G, H)** Overexpressing both GBP1 and GBP2 reduces the densities of ILP2 (G) and ILP5 (H) signals in the insulin-producing cells. The densities of ILP2 and ILP5 were quantified using ImageJ. We standardized the densities of ILPs by fixing the values from *C7*>GFP in the *gbp1*, *gbp2 ex67* mutant background to 1. *n* = 30–60. **(I)** Overexpressing both GBP1 and GBP2 reduces FRE-luciferase activity in the entire body of *gbp1*, *gbp2 ex67* mutant larvae. *n* = 5. Two copies of UAS transgenes were expressed using the *C7* Gal4 driver. For the wild-type control, we overexpressed two copies of UAS GFP using the *C7* Gal4 driver. Treatments sharing the same letter indicate the groups that are statistically indistinguishable from one another (ANOVA and pairwise *t* tests, *p* < 0.05). Growth rate was analyzed by ANCOVA and post hoc comparisons of the slopes. The supplementary file in which the data used to generate each plot can be found is S1 Data.

Next, we explored whether overexpressing GBP1 and GBP2 specifically in the fat body rescues the double null mutant phenotypes. Overexpressing both GBP1 and GBP2 in the fat body in the mutant background increased body size with respect to the null mutant controls (Fig 6A and 6B). The increase in body size was due to increased growth rate (Fig 6C). In addition, the duration of the L3 was slightly shortened in these animals (Fig 6D). These larvae showed less accumulation of ILPs (Fig 6E–6H), and lower FRE-luciferase activity than control animals (Fig 6I). These data suggest that the effects of GBP1 and GBP2 on body size could be mediated through their activity in the fat body.

### GBP1 and GBP2 Can Rescue the Effects of Inhibiting TOR Signaling in the Fat Body

**Fig 7:**
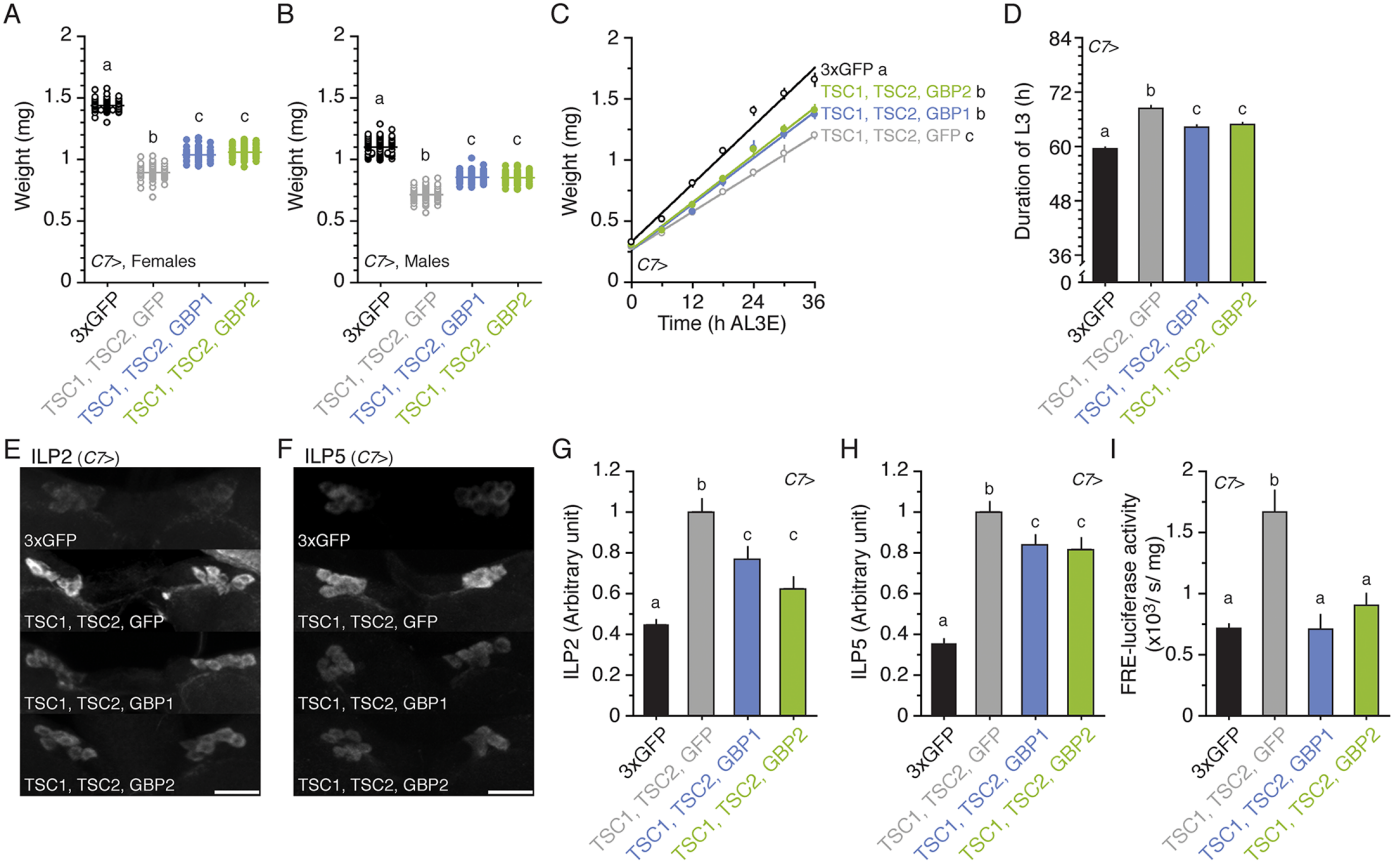
GBP1 and GBP2 are downstream of TOR signaling. **(A, B)** Overexpressing either GBP1 or GBP2 partially rescues body size reduction in females (A) and males (B) with reduced TOR signaling in their fat bodies. *n* = 47–64 for A and 59–67 for B. **(C)** Overexpressing either GBP1 or GBP2 partially increases growth rate in larvae with reduced TOR signaling in the fat body. *n* = 14-18/time point. **(D)** Overexpressing either GBP1 or GBP2 partially rescues the duration of the L3 in TOR signaling reduced larvae. *n* = 108–129. **(E, F)** Overexpressing either GBP1 or GBP2 reduces ILP2 (E) and ILP5 (F) accumulation in the insulin-producing cells. **(G, H)** Overexpressing GBP1 or GBP2 reduces the densities of ILP2 (G) and ILP5 (H) signals in the insulin-producing cells. We standardized the densities of ILPs by fixing the values from *C7*>GFP, TSC1, TSC2 to 1. *n* = 30–42 for both ILP2 and ILP5. **(I)** Overexpressing either GBP1 or GBP2 reduces FRE-luciferase activity in the entire body. *n* = 5. One copy of UAS transgene, UAS TSC1 and UAS TSC2 (three UAS in total) were co-expressed using the *C7* Gal4 driver for all experiments. For the wild-type control, we overexpressed three copies of UAS GFP using the *C7* Gal4 driver. Treatments sharing the same letter indicate the groups that are statistically indistinguishable from one another (ANOVA and pairwise *t* tests, *p* < 0.05). Growth rate was analyzed by ANCOVA and post hoc comparisons of the slopes. The supplementary file in which the data used to generate each plot can be found is S1 Data.

To analyze whether overexpressing either GBP1 or GBP2 is sufficient to rescue the effect of reduced TOR activity in the fat body, we co-overexpressed suppressors of TOR signaling, TSC1 and TSC2, and either GBP1 or GBP2 in the fat body. We found that overexpressing either GBP1 or GBP2 in the fat body partially relieved the body size reduction induced by TSC1/TSC2 overexpression (Fig 7A and 7B). Compared to larvae in which TSC1/TSC2 were overexpressed with GFP, larvae showed higher growth rates and shorter duration of the L3 if either GBP1 or GBP2 was also overexpressed (Fig 7C and 7D, respectively).

Finally, we determined whether GBP1 and GBP2 restored body size by increasing IIS activity. Compared with larvae overexpressing TSC1/TSC2 with GFP in the fat body, co-overexpressing TSC1/TSC2 with either GBP1 or GBP2 reduced ILP2 and ILP5 accumulation (Fig 7E–7H). In addition, overexpressing either GBP1 or GBP2 with TSC1/TSC2 in the fat body reduced FRE-luciferase activity (Fig 7I). Therefore, we conclude that GBP1 and GBP2 partially rescued the effect of reduced TOR activity in the fat body and that both GBP1 and GBP2 are downstream of TOR signaling in the fat body.

### GBP1 and GBP2 Directly Act on the Brain to Induce ILP2 and ILP5 Secretion

**Fig 8:**
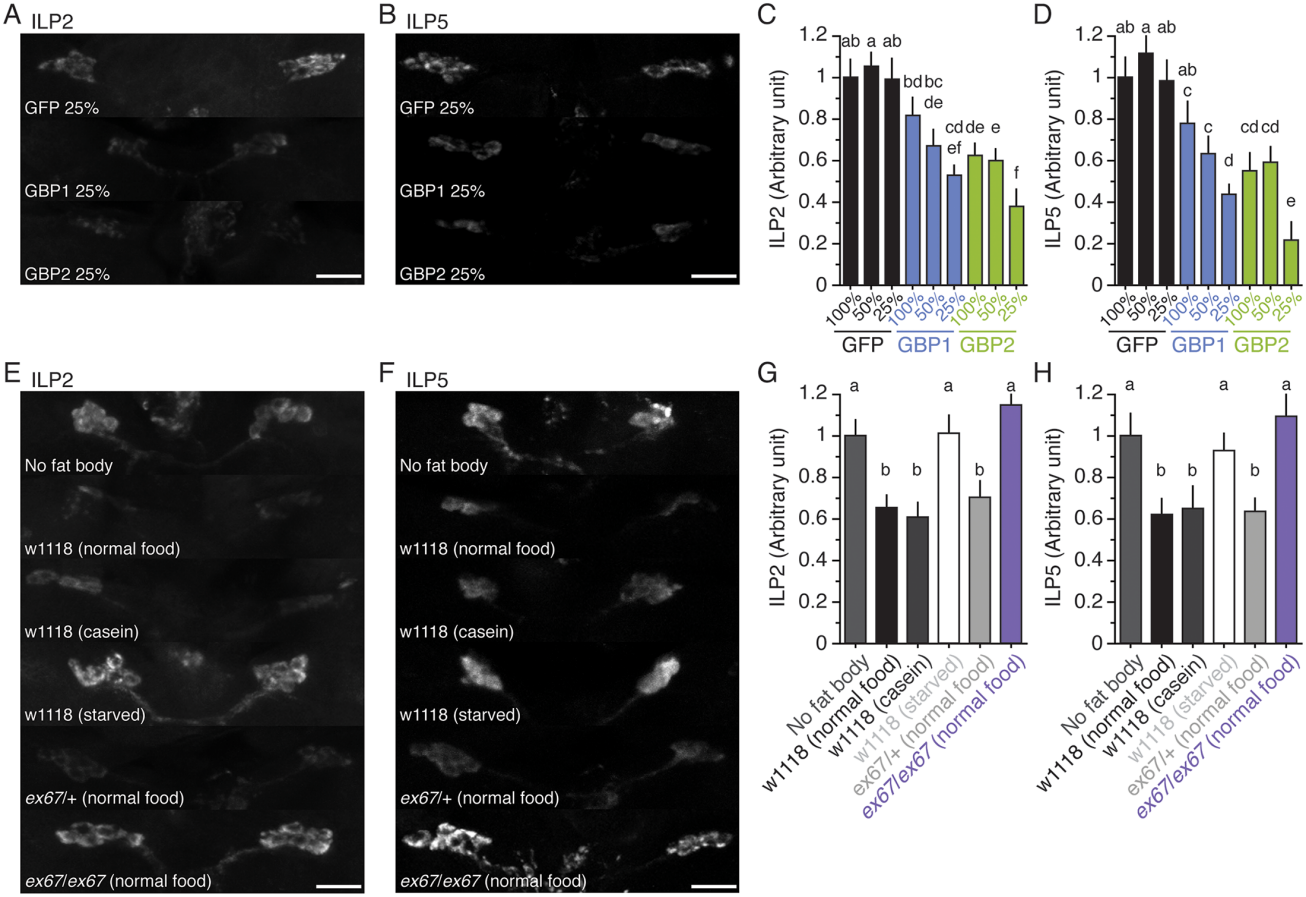
Both GBP1 and GBP2 are secreted from the fat body and directly act on the brain to induce ILP2 and ILP5 secretion. **(A, B)** Culturing wild-type brains with conditioned media from GBP1 and GBP2 expressing cells induces low level of ILP2 (A) and ILP5 (B) accumulation in the insulin-producing cells. To obtain brains, w1118 larvae staged at the onset of the L3 were fed on normal food for 12 h followed by 12 h incubation on 1% non-nutritive agar to induce strong ILP accumulation. To make conditioned media, an equal amount of Actin Gal4 plasmid and either UAS *gbp1*, UAS *gbp2*, or UAS *egfp* plasmid were transfected into the *Drosophila* SL2/DL2 cell line. Forty-eight hours after transfection, the media were centrifuged and the supernatant was diluted with culture medium to 100%, 50% and 25% the original concentrations. The scale bars are 20 μm. **(C, D)** Culturing wild-type brains with conditioned media from GBP1 and GBP2 expressing cells decreases the densities of ILP2 (C) and ILP5 (D) signals in the insulin-producing cells. We standardized the densities of ILPs by fixing the values from GFP 100% to 1. *n* = 16–27 for both ILP2 and ILP5. **(E, F)** Co-culturing wild-type brains with fat bodies from wild-type, but not from *gbp1*, *gbp2 ex67*, larvae induces low levels of ILP2 (E) and ILP5 (F) accumulation in the insulin-producing cells. The brains were obtained as above. The fat bodies were obtained as described in the Materials and Methods. **(G, H)** Co-culturing wild-type brains with fat bodies from wild-type, but not from *gbp1*, *gbp2 ex67*, larvae decreases the densities of ILP2 (G) and ILP5 (H) signals in the insulin-producing cells. We standardized the densities of ILPs by fixing the values from the “no fat body” treatment (culturing with a plain medium) to 1. *n* = 12–22 for both ILP2 and ILP5. Treatments sharing the same letter indicate the groups that are statistically indistinguishable from one another (ANOVA and pairwise *t* tests, *p* < 0.05). The supplementary file in which the data used to generate each plot can be found is S1 Data.

To explore whether GBP1 and GBP2 act directly on the brain to induce ILP secretion, we cultured the brains in vitro with the conditioned media from GBP1, GBP2 or GFP producing cells and assessed the levels of ILP2 and ILP5 accumulation. We tested for potential dose-dependent effects using three dilutions of this medium (25%, 50%, and 100%). Conditioned medium from both GBP1 and GBP2 expressing cells induced lower levels of ILP2 and ILP5 accumulation than GFP conditioned medium. Furthermore, the lowest dilutions of the GBP1 and GBP2 conditioned media, the 25% media, induced the lowest levels of ILP2 and ILP5 accumulation (Fig 8A–8D). Thus both GBP1 and GBP2 appear to act directly on the brain to induce ILP secretion in a dose-dependent manner.

We then co-cultured the wild-type brains with the fat bodies of *gbp1*, *gbp2 ex67* larvae, and assessed levels of ILP2 and ILP5 accumulation. First, we confirmed that the fat bodies from either normal food- or casein-fed larvae induced similar, lower levels of ILP2 and ILP5 accumulation (Fig 8E–8H). On the other hand, the fat bodies from starved wild-type larvae showed high levels of ILP2 and ILP5 accumulation, comparable to levels of accumulation in brains cultured in medium alone without fat body. Culturing wild-type brains with the fat bodies from fed *gbp1*, *gbp2 ex67* larvae showed high levels of ILP2 and ILP5 accumulation that were indistinguishable from those from brains cultured in medium alone or with starved fat bodies. Thus, we concluded that both GBP1 and GBP2 are secreted from the fat body in an amino acid-sensitive manner, and act on the brain directly to regulate ILP2 and ILP5 secretion. Whether or not GBP1 and GBP2 directly act on the insulin-producing cells or signal to a second set of cells in the brain to regulate insulin secretion is unclear.

## Discussion

Nutrition influences body size by affecting growth rate and the duration of the larval growth period. Exerting these effects requires communication between the organ that senses the nutritional status of the larvae, the fat body, and those that produce systemic hormones that regulate growth, the insulin-producing cells. Previously, the nature of this signal was unknown. Here, we provide evidence that GBP1 and GBP2 act as the signal that links nutrition sensing in the fat body with ILP secretion. We show that the mRNA expression of *gbp1* and *gbp2* is sensitive to dietary amino acids and TOR signaling in the fat body. Furthermore, these peptides control ILP secretion from the insulin-producing cells, which alters IIS activity in the entire body. Thus, our findings close an important gap in our understanding of how nutritional information is transmitted between the organs of the body.

Several studies show that the *Drosophila* fat body senses dietary nutrition and acts as a central regulator of growth and metabolism [10,15,26,38,39]. Once larvae feed, the fat body produces a number of signaling peptides, including growth factors and cytokines. Amongst these factors, the proposed fat-body–derived signals regulate ILP production and secretion in response to dietary protein. These signals not only regulate systemic growth and metabolism but also neuroblast reactivation by inducing ILP production in the glial cells [26,38,39]. Therefore, the fat body relays nutritional information to regulate a number of important developmental events.

Previous studies identified four distinct properties of the fat-body–derived signals. Our studies, together with results from other labs, show that GBP1 and GBP2 meet all four criteria. First, both GBP1 and GBP2 are produced in the larval fat body [18] and secreted into the hemolymph [18,19]. Furthermore, our in vitro brain co-culture experiments show that GBPs are secreted from the fat body and stimulate ILP2 and ILP5 secretion. Second, the mRNA expression of both *gbp1* and *gbp2* is sensitive to the protein content of the larval diet and to TOR signaling in the larval fat body. In addition, our rescue experiment revealed that reducing TOR activity in the fat body while simultaneously overexpressing either GBP1 or GBP2 in this tissue partially rescued body size, growth rate, and the duration of the L3. Furthermore, this manipulation stimulated an increase in ILP secretion when compared to larvae with reduced TOR activity in their fat bodies alone. Taken together, these results suggest that GBP1 and GBP2 act downstream of TOR signaling. Third, our co-culture experiments reveal that GBP1 and GBP2 act directly on the brain to regulate the secretion of ILPs by the insulin-producing cells. Our data does not allow us to distinguish whether they act on the insulin-producing cells themselves or on other cells in the brain to regulate ILP secretion. Finally, by regulating ILP secretion, both GBP1 and GBP2 affect systemic IIS signaling to regulate growth throughout the body. Taken together, our results provide strong evidence that both GBP1 and GBP2 act as fat-body–derived signals to regulate systemic growth by regulating ILP secretion in a nutrition-dependent manner. Whether GBPs are the only secreted factors capable of performing this function, and whether they are also able to regulate ILP secretion in other cells like the glial cells, remains to be discovered.

Our results further suggest that GBP1 and GBP2 do not play equal roles in regulating ILP secretion and systemic size regulation. Although we found that manipulating GBP2 expression accounts for most of the alterations in body size in response to decreased TOR signaling, GBP1 also appears to play a role since knocking down both peptides induces stronger size reductions. We found that accumulation of ILP2 was not enhanced by knocking down GBP2 alone, but accumulation of ILP2 increased when both GBP1 and GBP2 expression was reduced in the fat body. This suggests that GBP1 and GBP2 are at least partially redundant in function. Somewhat paradoxically, the expression level of GBP2 is lower than that of GBP1 in the fat body during the feeding stage. Nevertheless, our data suggest that GBP2 is the major signaling molecule produced in the fat body that regulates ILP secretion from the insulin-producing cells, with GBP1 playing a secondary role.

Although our work demonstrates that GBP1 and GBP2 provide systemic signals that regulate ILP secretion, we expect that other factors perform similar functions. Co-overexpressing GBP1 or GBP2 with TSC1, TSC2 did not completely rescue the body size phenotypes although TOR signaling regulates both *gbp1* and *gbp2*. This suggests that other factors produced in the fat body control ILP secretion from the insulin-producing cells. In fact, Unpaired 2 and CCHamide-2 produced in the fat body regulate ILP secretion from the insulin-producing cells, although their expression is regulated by carbohydrates and lipids instead of amino acids [14,15]. Perhaps, the interplay between different macronutrients is important for expression of these signals. Further studies are needed to elucidate this issue.

Interestingly, we found two slightly inconsistent results when we compared the phenotypes of larvae in which both GBP1 and GBP2 were knocked down in the fat body and the *gbp1*, *gbp2 ex67* mutant larvae. Although there was no effect in the null mutant, we found *ilp2* mRNA expression increased and AKT expression level was down regulated in larvae where either TOR signaling activity or the expression of GBP1 and GBP2 was specifically reduced in the fat body. These differences might be due to the difference between a null mutation, which eliminates all expression of the gene, and partial reduction of expression of GBP1 and GBP2 via RNAi. For instance, very small concentrations of GBPs might either directly or indirectly act on the insulin-producing cells to regulate *ilp2* mRNA expression and peripheral tissues to regulate AKT expression. Alternatively, it is also possible that GBP expression in other organs interacts with the GBP expression in the fat body to reduce some of its effects. Further precise studies are necessary to resolve this problem.

GBP was originally identified in lepidopterans as a bipolar growth regulator; low concentrations of GBP enhance growth and cell proliferation, but high concentrations suppress growth [16,17]. We found that overexpressing Rheb in the fat body resulted in slightly smaller body size, as it reduces the duration of the L3. Furthermore, overexpressing either GBP1 or GBP2 induced a slight increase in body size at 25°C, but a greater increase in size at 22°C. Since we know the Gal4-UAS system is sensitive to temperature and in fact, expression levels of these transgenes differ at 22°C and 25°C, *Drosophila* GBPs might also suppress ILP secretion from the insulin-producing cells when expressed at high concentrations. In addition, brains cultured with the conditioned media from GBP1 or GBP2 expressing cells revealed that diluted media induced ILP2 and ILP5 secretion more effectively. Taken together, the effects of GBP1 and GBP2 appear dose-dependent. The mechanism through which these peptides exert these effects requires further dedicated studies.

Nutrition does not merely affect insect body size, but affects a wide range of health and fitness of multicellular organisms. In mammals, leptin is secreted from the adipose tissue and acts systemically to control feeding behavior and metabolism [40]. Loss of leptin function results in several metabolic phenotypes, including obesity in mammals [41]. Interestingly, a loss of function of Unpaired 2 in *Drosophila* is rescued by overexpressing mammalian leptin [15]. Also, EGF, a mammalian ortholog of GBPs, regulates a half-life of Survivin to regulate beta-cell expansion, which is required to maintain metabolic homeostasis [42]. Given these parallels between mammals and insects, we expect our findings in *Drosophila* to provide a framework for understanding the inter-organ communication that allows nutrient sensing organs to coordinate insulin secretion with the nutritional environment in a broad range of animals.

## Materials and Methods

### *Drosophila* Strains, Construct Preparation, and Transgenic Fly Generation

For GBP1, GBP2 and GBP3 knockdown experiments, we used Vienna Drosophila RNAi Center 108755, 16696 and 6838, respectively. To manipulate TOR signaling, we used w; UAS TSC1, UAS TSC2 and w; UAS Rheb (gifts from Dr. Carlos Ribeiro, Champalimaud Centre for the Unknown). The G-TRACE experiment to evaluate Gal4 expression throughout the embryonic and larval stages in *C7* Gal4 lines was carried out using; w*; P{UAS-RedStinger}4, P{UAS-FLP.D}JD1, P{Ubi-p63E(FRT.STOP)Stinger}9F6/ CyO (Bloomington Drosophila Stock Center #28280, a gift from Dr. Alexandre B Leitão and Mr. Julien Marcetteau, Instituto Gulbenkian de Ciência). To prepare UAS overexpression constructs, the entire open reading frames of GBP1 and GBP2 were isolated by RT-PCR using cDNA made from w1118 wandering larvae with primer sets as described in S1 Table. After PCR products were cloned and sequenced, the constructs were inserted into pUAST attB vector using *Bgl*II and *Xba*I, then integrated into the third chromosome by site-directed insertion using the phiC31 integrase and an attP2 landing site carrying recipient line, y,w, P{y[+].nos-int.NLS}; P{CaryP}attP2 (a gift from Dr. Diogo Manoel, Instituto Gulbenkian de Ciência). We created the *gbp1*, *gbp2 ex67* mutant by a P-element excision mutagenesis method using the P-element insertion line, y[1] w[*]; P{w[+mC] = EP}G19340 (Bloomington Drosophila Stock Center #28111). The *gbp1*, *gbp2 ex67* mutant was backcrossed three times to w1118 line.

### Staging, Nutritional Manipulations, and Weight Study

For all experiments, larvae were reared at controlled densities (200 larvae/60 mm diameter food plate or 30 larvae/vial). Eggs were collected every 4 h as described previously [27]. Newly molted L3 larvae were collected every 2 h as described previously [27,28]. Collected larvae were raised in a standard cornmeal/molasses medium without additional yeast until the desired time point at 25°C, unless mentioned. For starvation treatments, we used 1% non-nutritive agar without any additives. For nutritional manipulations, we used either 20% sucrose, 5% linseed oil or 5% enzymatic digested casein (Sigma) in 1% non-nutritive agar as sources of sugar, lipid and amino acids, respectively. To measure final body size, we weighed pharate adults, which are approximately 2–14 h before eclosion. We separated males and females based on the presence or absence of sex combs. We carefully washed all individuals with distilled water and a paintbrush, and then dried them for at least 15 min on paper towels before weighing them. We weighed individual pupae using an ultra-microbalance (Sartorius, SE2). To determine the duration of the L3, we staged larvae at the onset of the L3, and observed pupariation time every 2 h.

### Western Blot Analysis

We extracted protein from wing discs of 24 h AL3E larvae for pAKT western blot analyses. The discs were dissected in ice-cold PBS, and homogenized in NB Buffer (150 mM NaCl, 50 mM Tris–HCl pH 7.5, 2 mM EDTA, 0,1% NP-40, 1 mM DTT, 10 mM NaF) with Complete protease inhibitor cocktail and PhosSTOP phosphatase inhibitor cocktail (Roche). After protein concentrations were quantified using a BCA assay, we loaded equal amount of samples with 2x loading buffer. We used the following antibodies: anti-Phospho-Drosophila Akt (Ser505) antibody (1:1,000, Cell Signaling), anti-Akt (pan) antibody (1:1,000, Cell Signaling) and anti-Histone H3 (1:1,000, Cell Signaling). For an ILP2 western blot analysis, we extracted hemolymph from 24 h AL3E larvae. The hemolymph was extracted on ice. After centrifugation at 16,000 g for 5 min at 4°C, supernatant was heat-inactivated at 60°C for 10 min followed by additional centrifugation at 16,000 g for 5 min at 4°C. After we added EDTA, NP-40, DTT, NaF and Complete protease inhibitor cocktail, equal amount of 2x loading buffer was added and kept at -80°C until use. We used a Tris-Glycine gel and an anti-ILP2 antibody (1:8,000, a gift from Dr. Pierre Léopold) [10].

### Immunocytochemistry

Immunocytochemistry was performed using standard methods as described previously [27,43]. All brains were carefully staged and dissected at 24 h AL3E. The antibodies we used were: anti-ILP2 (1:800) and anti-ILP5 (1:800, a gift from Dr. Pierre Léopold). Specimens were imaged using a Zeiss Meta 510 confocal microscope maintaining the same imaging settings for each set of experiments. Images were processed using ImageJ and Adobe Photoshop. The densities of ILP2 and ILP5 were quantified using ImageJ.

### Quantitative PCR (qPCR)

Total RNA was extracted using TRIzol (Invitrogen) from either the fat body or whole body. After DNase treatment, total RNA concentration was quantified and 1 μg total RNA was converted to cDNA using oligo dT and reverse transcriptase. qPCR was performed using SYBR Green PCR Master Mix (Applied Biosystems) and ABI 7900HT (Applied Biosystems). The primers used for this study are listed in S1 Table [27,44].

### FRE-Luciferase Assays

The FRE-luciferase transgenic line [34] is a gift from Dr. Alexander W. Shingleton (Lake Forest College, IL). FRE-luciferase assays were performed as described previously [35] with a minor modification. Briefly, we staged larvae at the onset of the L3 and collected them at 24 h AL3E. Larvae were washed with distilled water twice, and stored at -80°C until use. We used five larvae per sample and examined five replicates per genotype. Protein was extracted into 200 μl of Luciferase Cell Culture Lysis Reagent (Promega), and 40 μl of protein extract was used for luciferase assays. Luciferase assays were performed using the Luciferase Assay System (Promega) according to the manufacturer's instructions. We measured the protein concentration for each sample using a BCA assay and normalized the luciferase activity per milligram of protein.

### In Vitro Brain Co-culture with the Fat Bodies and Culture with Conditioned Media

The brains were cultured according to Britton and Edgar [38] and Geminard *et al*. [10] with minor modifications. To obtain brains, w1118 larvae staged at the onset of the L3 as described above were fed on normal food for 12 h followed by 12 h incubation on 1% non-nutritive agar to induce strong ILP accumulation. To obtain fat bodies, we also used carefully staged 24 h AL3E w1118, *gbp1*, *gbp2 ex67* heterozygote, and *gbp1*, *gbp2 ex67* homozygote larvae. To obtain the fat bodies from casein treated or starved larvae, we fed w1118 larvae on normal food until 12 h AL3E and then transferred them into 5% casein food or 1% non-nutritive agar, respectively, and incubated for additional 12 h. Larvae were surface-sterilized in 70% ethanol for a few minutes, rinsed twice in sterilized distilled water, and dissected in a Schneider’s *Drosophila* medium (Biowest) with 10% FBS (Gibco) and 1% Penicillin-Streptomycin (Sigma). All co-culture experiments were done in a 1.5 ml microcentrifuge tube with a 20 μl of Schneider’s medium with 10% FBS (Gibco) and 1% Penicillin-Streptomycin for 4 h at 22°C. Carefully dissected organs were transferred into the media using a fine dissecting needle to minimize the volume of medium transferred. Five brains were co-cultured with 15 fat bodies. To avoid contact between the tissues, we ensured that the dissected brains sunk to the bottom of the tube and that the fat bodies were floating on top of the media. To prepare conditioned media, we used the *Drosophila* SL2/DL2 cell line. For transfection, the cells were cultured in Schneider’s *Drosophila* medium with 10% FBS without any other additives. The cells were transfected with equal amount of Actin Gal4 plasmid (a gift from Dr. Catarina Brás-Pereira, Instituto Gulbenkian de Ciência) and either UAS *gbp1*, UAS *gbp2* or UAS *egfp* plasmid using FuGENE HD Transfection Reagent (Promega) according to the manufacturers instructions. Forty-eight hours after transfection, the media were centrifuged at 800 *g* for 3 min and the supernatant was transferred into new tubes. After we confirmed there were no live cells in the media under a microscope, we used the conditioned media to culture brains from w1118 larvae as mentioned above.

### Statistical Analysis

Data analysis was performed in R (www.r-project.org) and in KaleidaGraph (Synergy), and the plots were generated in KaleidaGraph. We tested for differences in male and female body size, L3 duration, relative expression of *gbp1* and *gbp2*, ILP2 and ILP5 accumulation, pAKT/HH3 and pAKT/AKT ratios, and FRE luciferase activity using ANOVA and post hoc pairwise *t* tests. To test for differences in growth rates between genotypes, we fit the data using linear models regressing larval weight against age and tested for differences in the interaction term between larval age and genotype using ANCOVA and post hoc comparisons of the slopes of fitted lines using lstrends (HH and lsmeans packages).

## Supporting Information

S1 Data Supporting data. (XLSX)

S1 Fig The *C7* Gal4 driver shows strong Gal4 expression in the fat body. **(A)** Gal4 lineage-traced and live Gal4 expression in the *C7* Gal4 using the G-TRACE method. GFP indicates lineage-traced Gal4 expression, DsRed indicates live Gal4 expression and phalloidin indicates cellular shape. All organs were dissected from the same 24 h AL3E larva except for hemocytes. Hemocytes were collected from six 24 h AL3E larvae. **(B)** Quantification of *gal4*, *gbp1*, and *gbp2* mRNA in *C7* Gal4 larvae. We normalized the values using an internal control, *RpL3*. Then, we standardized the expression level of each gene by fixing the values in the fat body to 1. We used five organs from 24 h AL3E larvae for each sample and three biologically independent samples for each organ. Each bar indicates the relative mean expression ± SEM. The supplementary file in which the data used to generate each plot can be found is S1 Data.
(TIF)

S2 Fig The effects of RNAi-mediated knock down on *gbp1*, *gbp2*, *and gbp3* mRNA expression. We overexpressed either GFP or RNAi lines using the *C7* Gal4 driver in the fat body and quantified mRNA expression of *gbp1* (A), *gbp2* (B) and *gbp3* (C) in the whole larvae. We normalized the values using an internal control, *RpL3*. Then, we standardized the expression level of each gene by fixing the values from *C7*>GFP larvae to 1. We used five larvae for each sample and three biologically independent samples for each condition. The supplementary file in which the data used to generate each plot can be found is S1 Data.
(TIF)

S3 Fig *gbp1* expression is stronger than *gbp2* in the fat body. **(A, B)** Expression profile of *gbp1* (A) and *gbp2* (B) in the whole larvae. We carried out qPCR using w1118. We normalized the values using an internal control, *RpL3*. Then, we standardized the expression level of each gene by fixing the values 0 h AL3E larvae to 1 in A and B. We used five larvae for each sample and three biologically independent samples for each condition. **(C)** Semi-quantitative PCR shows *gbp1* expression is dominant in the fat body. Total RNA was extracted from the fat body of 24 h AL3E larvae and the same amount of cDNA was used for RT-PCR. Three independent specimens (a, b, and c) were used for PCR. Cycle number is 28 for both *gbp1* and *gbp2*. The supplementary file in which the data used to generate each plot can be found is S1 Data.
(TIF)

S4 Fig Reducing GBP1 and GBP2 expression in the fat body reduces body size due to reduced growth rate. **(A, B)** GBP1, GBP2 double-knockdown in the fat body reduces final body size in females (A) and males (B). *n* = 78–98 for A and *n =* 66–100 for B. **(C)** GBP1, GBP2 double-knockdown in the fat body reduces growth rate. *n =* 13–17/time point. **(D)** GBP1, GBP2 double-knockdown extends the duration of the L3. *n* = 165–169. To compensate the effect of transgenes, we overexpressed two copies of UAS transgenes using the *ppl* Gal4 driver. Treatments sharing the same letter indicate the groups that are statistically indistinguishable from one another (ANOVA and pairwise *t* tests, *p* < 0.05). Growth rate was analyzed by ANCOVA and post hoc comparisons of the slopes. The supplementary file in which the data used to generate each plot can be found is S1 Data.
(TIF)

S5 Fig GBP1 and GBP2 regulate ILP2 and ILP5 secretion. **(A, B)** Knocking down GBP1 and GBP2 increases ILP2 (A) and ILP5 (B) accumulation in the insulin-producing cells. The scale bars are 20 μm. **(C, D)** Knocking down GBP1 and GBP2 increases the densities of ILP2 (C) and ILP5 (D) signals in the insulin-producing cells. We standardized the densities of ILPs by fixing the values from *ppl*>2xGFP to 1. *n* = 31–46 for both ILP2 and ILP5. Treatments sharing the same letter indicate the groups that are statistically indistinguishable from one another (ANOVA and pairwise *t* tests, *p* < 0.05). The supplementary file in which the data used to generate each plot can be found is S1 Data.
(TIF)

S6 Fig Reducing GBP1 and GBP2 expression alters *ilp2* and *ilp5* mRNA expression. **(A, B)** GBP1, GBP2 double-knockdown in the fat body increases *ilp2* expression (A) but reduces *ilp5* expression (B). To control for transgene copy number, we overexpressed two copies of UAS transgenes using the *C7* Gal4 driver. **(C, D)** The *gbp1*, *gbp2 ex67* mutant shows no effect on *ilp2* expression (C) but decreases *ilp5* expression (D). We normalized the values using an internal control, *RpL3*. Then, we standardized the expression level of each gene by fixing the values from *C7*>2xGFP or w1118 animals to 1. We used five larvae for each sample and five biologically independent samples for each genotype. Each bar indicates the relative mean expression ± SEM. Treatments sharing the same letter indicate the groups that are statistically indistinguishable from one another (ANOVA and pairwise *t* tests, *p* < 0.05). The supplementary file in which the data used to generate each plot can be found is S1 Data.
(TIF)

S7 Fig Milder overexpression of GBP1 and GBP2 shows stronger effect on growth. **(A-D)** Overexpressing GBP1 or GBP2 increases body size in wild-type females and males (A and B, respectively) at 25°C or 22°C (C and D, respectively). One copy of UAS transgenes was expressed using the *C7* Gal4 driver. *n* = 70 for A, 69–72 for B, 61–74 for C, and 59–68 for D. **(E, F)** Expression level of GBP1 (E) or GBP2 (F) at 25°C is higher than that of 22°C. We normalized the values using an internal control, *RpL3*. Then, we standardized the expression level of each gene by fixing the values from *C7*>GFP at 25°C to 1. We used five larvae for each sample and five biologically independent samples for each genotype. Each bar indicates the relative mean expression ± SEM. **(G, H)** Overexpressing GBP1 or GBP2 at two different temperatures shows reduced ILP2 (G) and ILP5 (H) accumulation in the insulin-producing cells. The scale bars are 20 μm. **(I, J)** Overexpressing GBP1 or GBP2 at two different temperatures shows reduced densities of ILP2 (I) and ILP5 (J) signals in the insulin-producing cells. We standardized the densities of ILPs by fixing the values from *C7*>GFP at 25°C to 1. *n* = 19–46 for both ILP2 and ILP5. Treatments sharing the same letter indicate the groups that are statistically indistinguishable from one another (ANOVA and pairwise *t* tests, *p* < 0.05). The supplementary file in which the data used to generate each plot can be found is S1 Data.
(TIF)

S1 Table Primer list. (DOCX)

## Abbreviations

AL3E: after L3 ecdysis
FoxO: Forkhead Box O
FRE: FoxO-Response Element
GBP: Growth-Blocking Peptide
GFP: green fluorescent protein
IIS: insulin/insulin-like growth factor signaling
ILP: insulin-like peptide
L3: third larval instar
pAKT: phosphorylated AKT
ppl: pumpless
qPCR: quantitative PCR
Rheb: Ras homolog enriched in brain ortholog
TOR: Target of Rapamycin
