# Correction: Growth-Blocking Peptides As Nutrition-Sensitive Signals for Insulin Secretion and Body Size Regulation

**DOI:** 10.1371/journal.pbio.1002551

**Published:** 2016-08-30

**Authors:** Takashi Koyama, Christen K. Mirth

In the original version of the article, the middle image of Panel F in Fig 6 was accidentally duplicated. The correct version of Fig 6 can be found below. The figure legend and conclusion remain the same.

**Fig 6:**
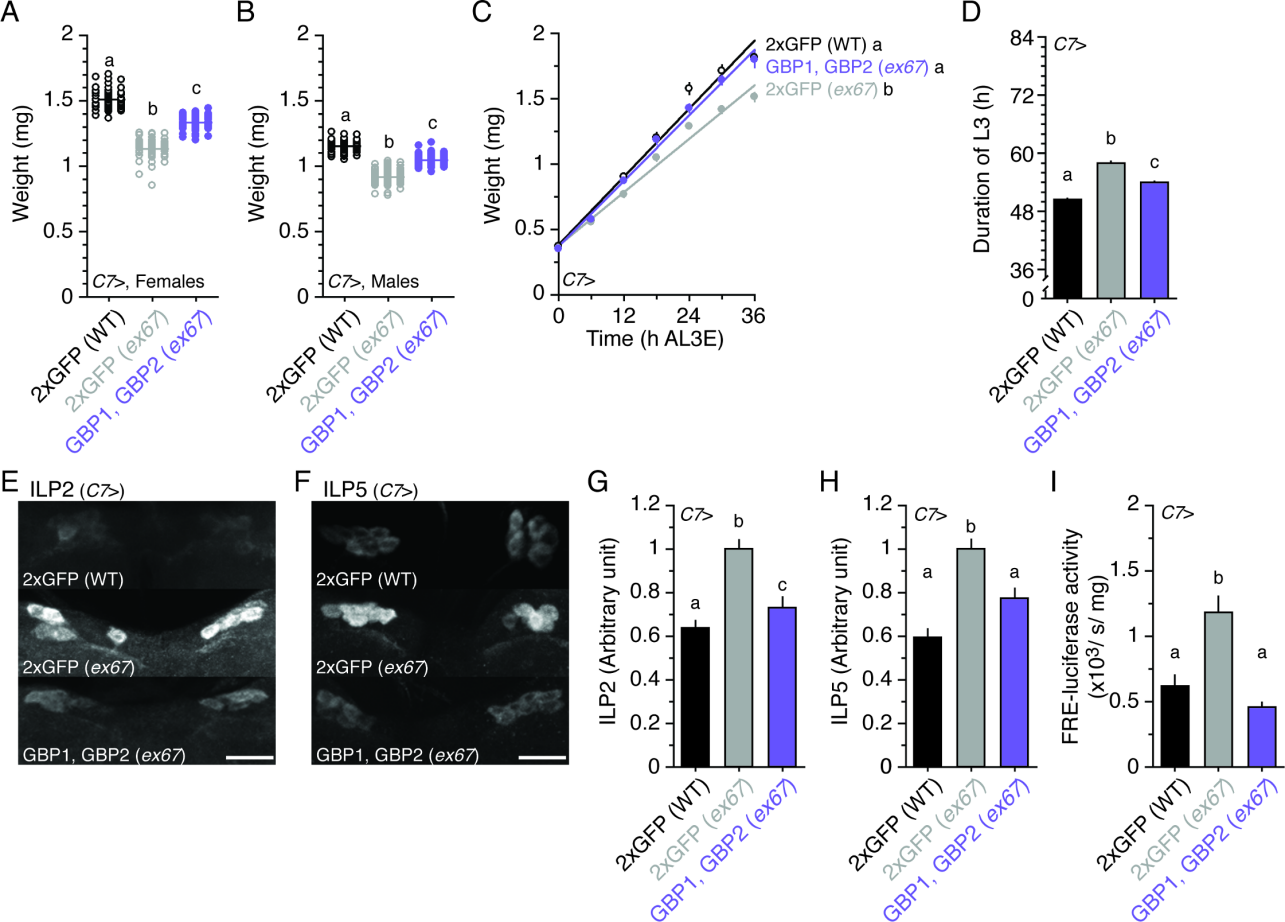
Overexpressing GBP1 and GBP2 in the fat body rescues body size in *gbp1*, *gbp2 ex67* null mutant larvae. **(A, B)** Overexpressing both GBP1 and GBP2 partially rescues body size reduction in *gbp1*, *gbp2 ex67* mutant females (A) and males (B) at 22°C. *n* = 51–52 for A and *n* = 54–65 for B. **(C)** Overexpressing both GBP1 and GBP2 partially increases growth rate in *gbp1*, *gbp2 ex67* mutant larvae. *n* = 14–20/time point. **(D)**Overexpressing both GBP1 and GBP2 partially rescues the duration of the L3 in *gbp1*, *gbp2 ex67* mutant larvae. *n* = 111–119. **(E, F)** Overexpressing both GBP1 and GBP2 reduces ILP2 (E) and ILP5 (F) accumulation in the insulin-producing cells of *gbp1*, *gbp2 ex67* mutant larvae. Larvae were staged at the onset of the L3, and then fed on normal food for 24 h. The insulin-producing cells were immunostained using an anti-ILP2 antibody and an anti-ILP5 antibody. **(G, H)** Overexpressing both GBP1 and GBP2 reduces the densities of ILP2 (G) and ILP5 (H) signals in the insulin-producing cells. The densities of ILP2 and ILP5 were quantified using ImageJ. We standardized the densities of ILPs by fixing the values from *C7*>GFP in the *gbp1*, *gbp2 ex67* mutant background to 1. *n* = 30–60. **(I)** Overexpressing both GBP1 and GBP2 reduces FRE-luciferase activity in the entire body of *gbp1*, *gbp2 ex67* mutant larvae. *n* = 5. Two copies of UAS transgenes were expressed using the *C7* Gal4 driver. For the wild-type control, we overexpressed two copies of UAS GFP using the *C7* Gal4 driver. Treatments sharing the same letter indicate the groups that are statistically indistinguishable from one another (ANOVA and pairwise *t* tests, *p* < 0.05). Growth rate was analyzed by ANCOVA and post hoc comparisons of the slopes. The supplementary file in which the data used to generate each plot can be found is S1 Data.

## References

[1] Koyama T, Mirth CK (2016) Growth-Blocking Peptides As Nutrition-Sensitive Signals for Insulin Secretion and Body Size Regulation. PLoS Biol 14(2): e1002392 doi:10.1371/journal.pbio.1002392 26928023 10.1371/journal.pbio.1002392 PMC4771208

