# Supplementary figures and images for: Growth-Blocking Peptides As Nutrition-Sensitive Signals for Insulin Secretion and Body Size Regulation
Source: PLoS Biol. 2016 Feb 29;14(2):e1002392. doi: 10.1371/journal.pbio.1002392 (PMC4771208; doi:10.1371/journal.pbio.1002392)

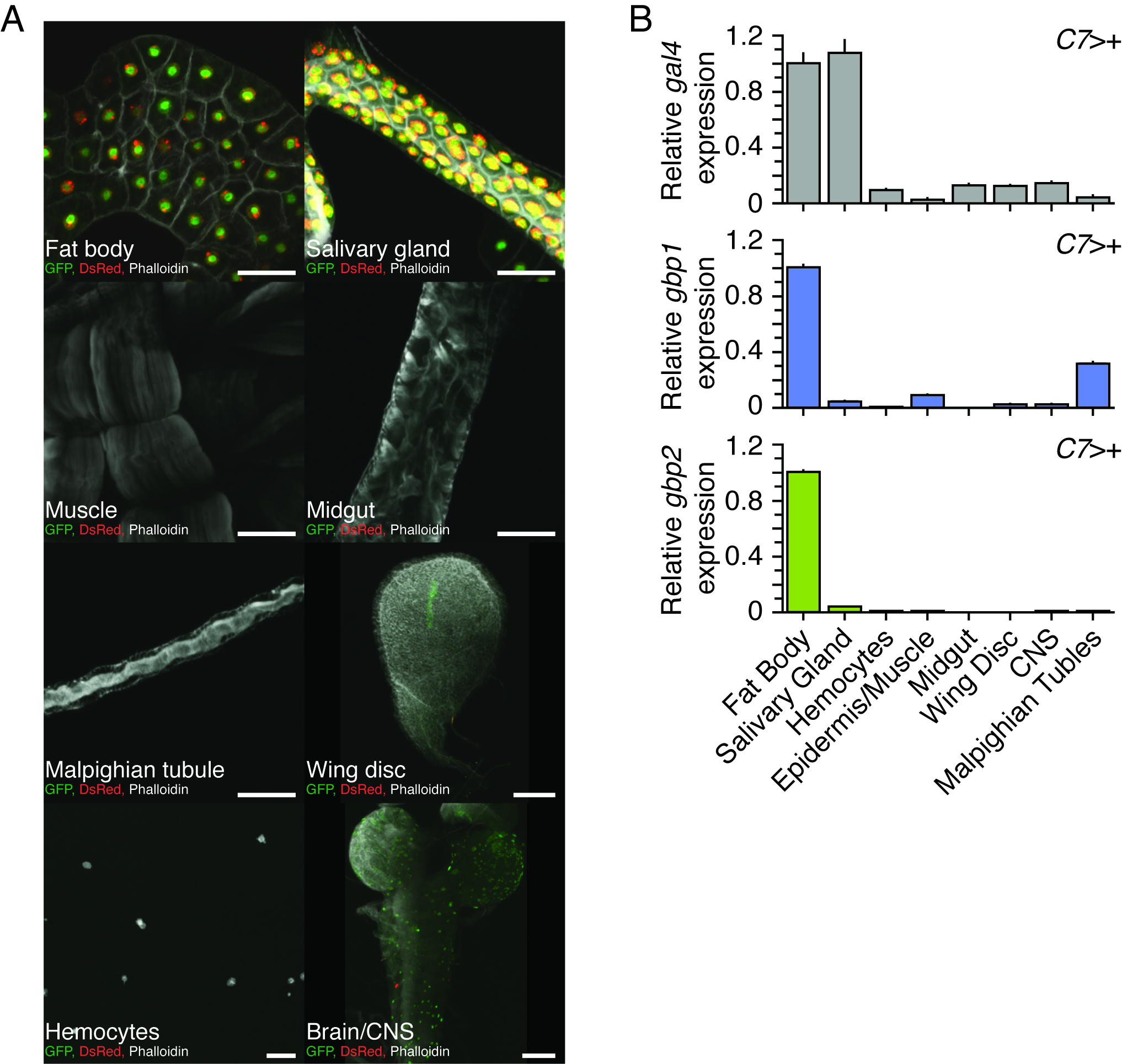

Supplement: S1 Fig — (A) Gal4 lineage-traced and live Gal4 expression in the C7 Gal4 using the G-TRACE method. GFP indicates lineage-traced Gal4 expression, DsRed indicates live Gal4 expression and phalloidin indicates cellular shape. All organs were dissected from the same 24 h AL3E larva except for hemocytes. Hemocytes were collected from six 24 h AL3E larvae. (B) Quantification of gal4, gbp1, and gbp2 mRNA in C7 Gal4 larvae. We normalized the values using an internal control, RpL3. Then, we standardized the expression level of each gene by fixing the values in the fat body to 1. We used five organs from 24 h AL3E larvae for each sample and three biologically independent samples for each organ. Each bar indicates the relative mean expression ± SEM. The supplementary file in which the data used to generate each plot can be found is S1 Data. (TIF) [file pbio.1002392.s002.tif]

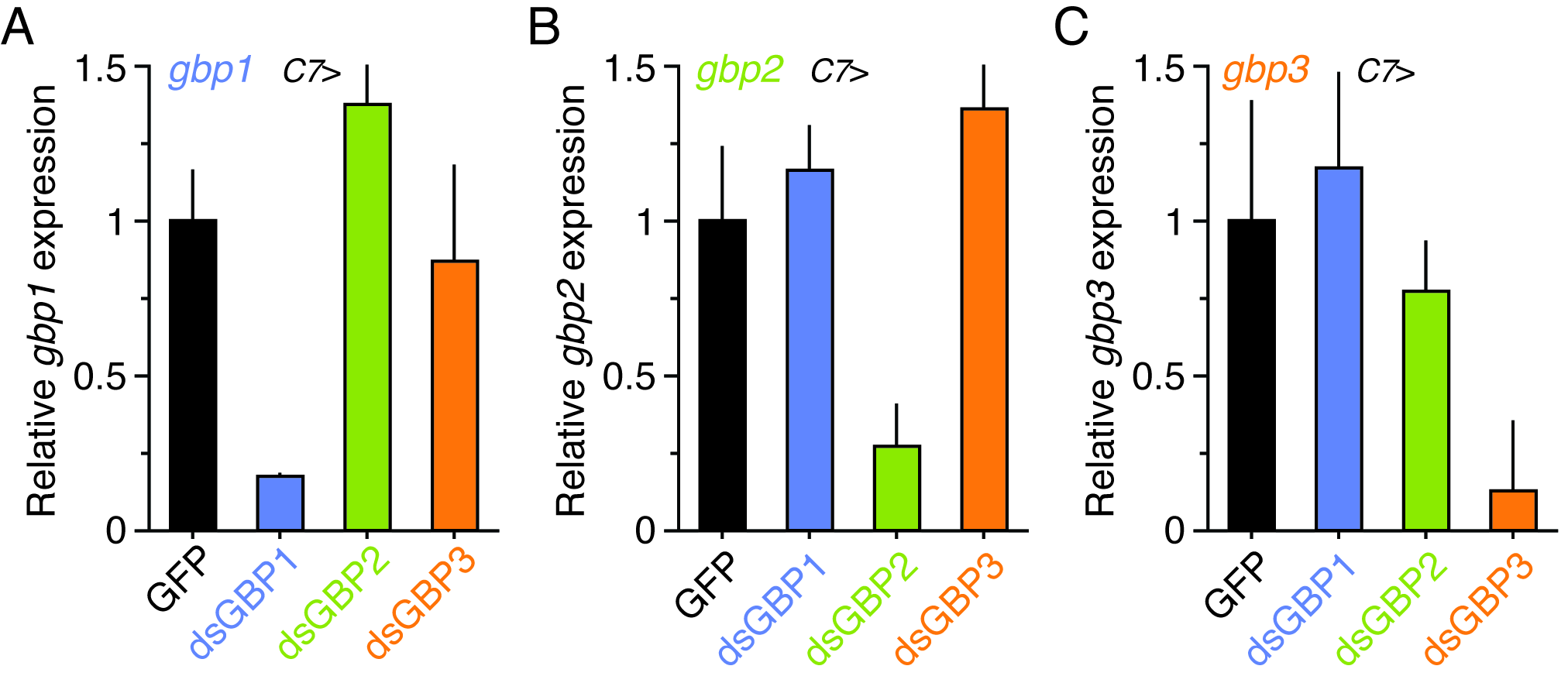

Supplement: S2 Fig — We overexpressed either GFP or RNAi lines using the C7 Gal4 driver in the fat body and quantified mRNA expression of gbp1 (A), gbp2 (B) and gbp3 (C) in the whole larvae. We normalized the values using an internal control, RpL3. Then, we standardized the expression level of each gene by fixing the values from C7>GFP larvae to 1. We used five larvae for each sample and three biologically independent samples for each condition. The supplementary file in which the data used to generate each plot can be found is S1 Data. (TIF) [file pbio.1002392.s003.tif]

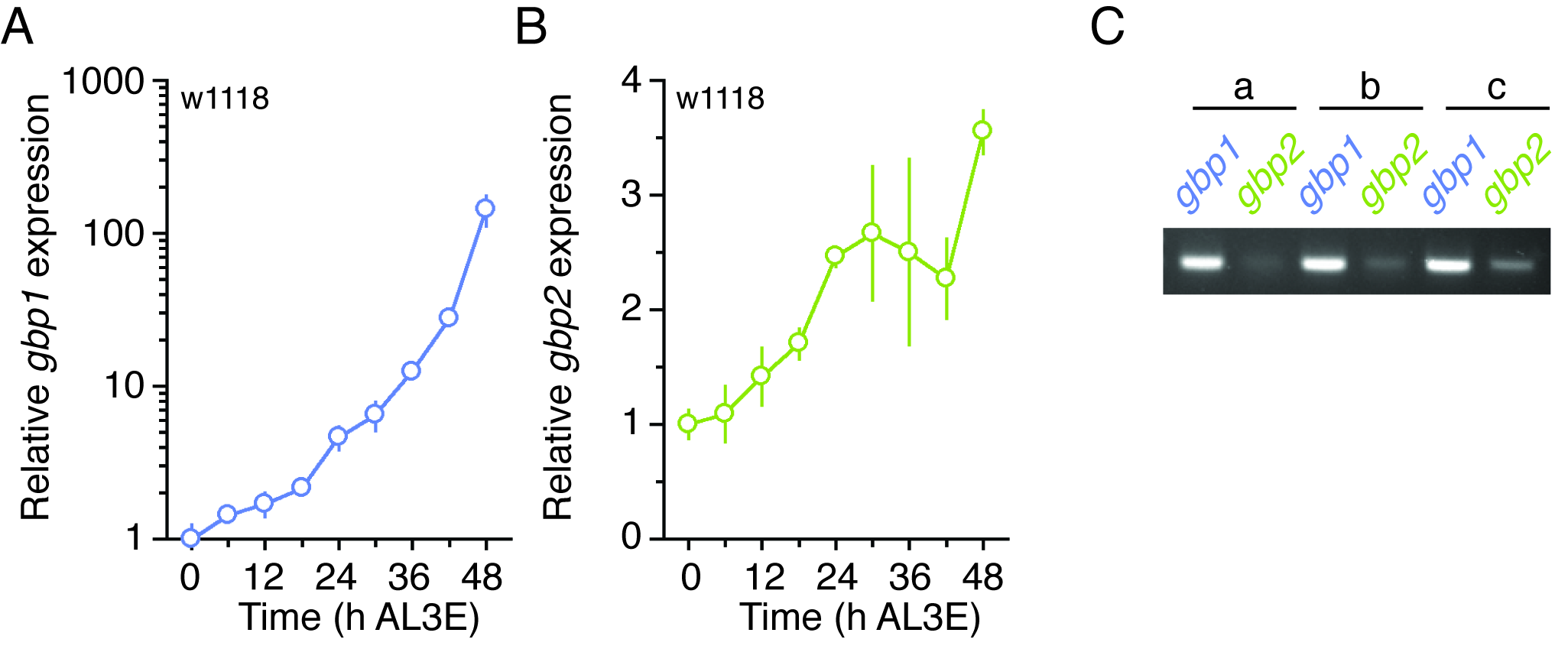

Supplement: S3 Fig — (A, B) Expression profile of gbp1 (A) and gbp2 (B) in the whole larvae. We carried out qPCR using w1118. We normalized the values using an internal control, RpL3. Then, we standardized the expression level of each gene by fixing the values 0 h AL3E larvae to 1 in A and B. We used five larvae for each sample and three biologically independent samples for each condition. (C) Semi-quantitative PCR shows gbp1 expression is dominant in the fat body. Total RNA was extracted from the fat body of 24 h AL3E larvae and the same amount of cDNA was used for RT-PCR. Three independent specimens (a, b, and c) were used for PCR. Cycle number is 28 for both gbp1 and gbp2. The supplementary file in which the data used to generate each plot can be found is S1 Data. (TIF) [file pbio.1002392.s004.tif]

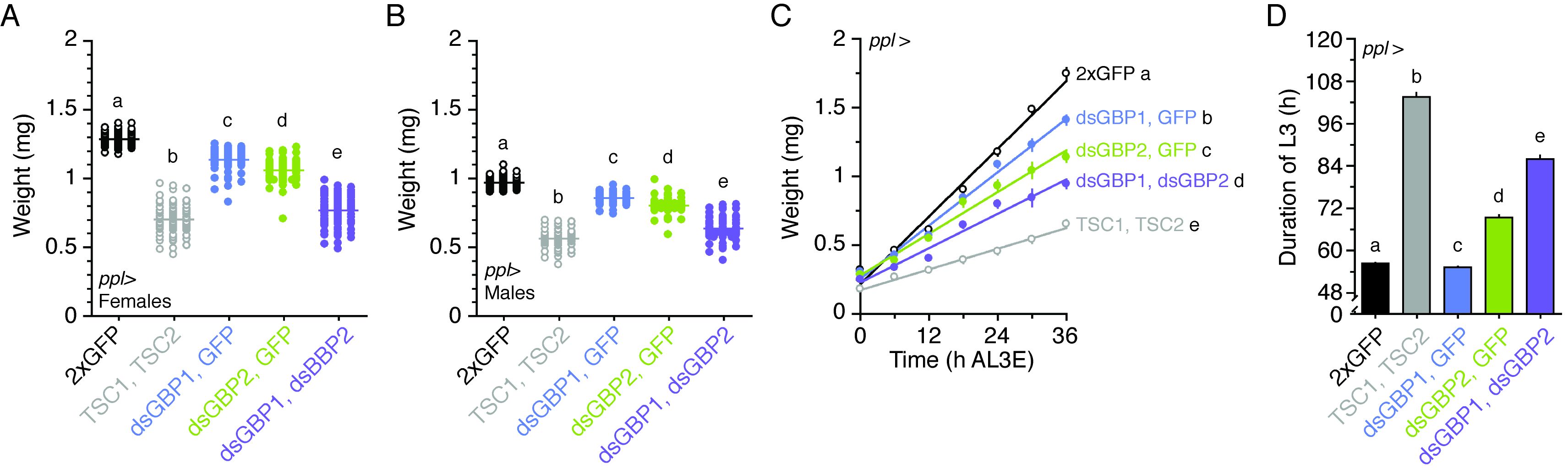

Supplement: S4 Fig — (A, B) GBP1, GBP2 double-knockdown in the fat body reduces final body size in females (A) and males (B). n = 78–98 for A and n = 66–100 for B. (C) GBP1, GBP2 double-knockdown in the fat body reduces growth rate. n = 13–17/time point. (D) GBP1, GBP2 double-knockdown extends the duration of the L3. n = 165–169. To compensate the effect of transgenes, we overexpressed two copies of UAS transgenes using the ppl Gal4 driver. Treatments sharing the same letter indicate the groups that are statistically indistinguishable from one another (ANOVA and pairwise t tests, p < 0.05). Growth rate was analyzed by ANCOVA and post hoc comparisons of the slopes. The supplementary file in which the data used to generate each plot can be found is S1 Data. (TIF) [file pbio.1002392.s005.tif]

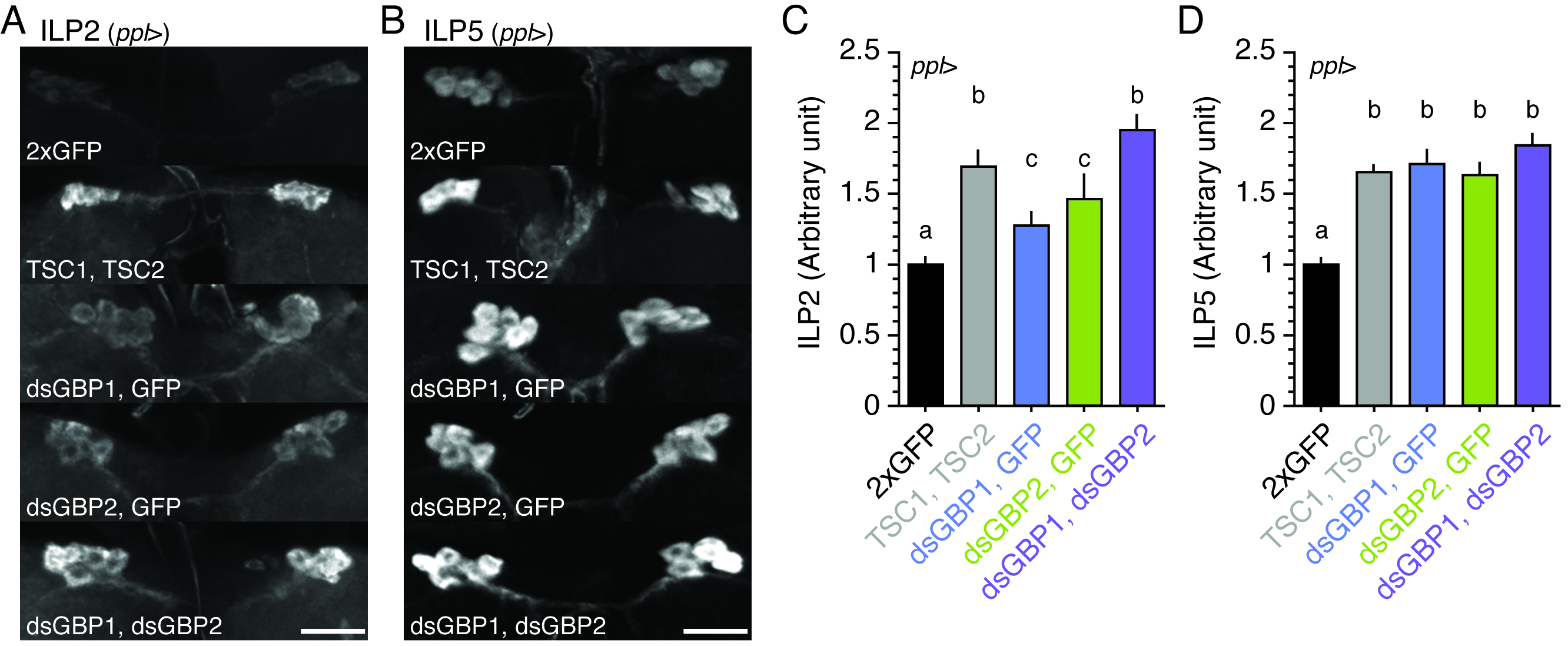

Supplement: S5 Fig — (A, B) Knocking down GBP1 and GBP2 increases ILP2 (A) and ILP5 (B) accumulation in the insulin-producing cells. The scale bars are 20 μm. (C, D) Knocking down GBP1 and GBP2 increases the densities of ILP2 (C) and ILP5 (D) signals in the insulin-producing cells. We standardized the densities of ILPs by fixing the values from ppl>2xGFP to 1. n = 31–46 for both ILP2 and ILP5. Treatments sharing the same letter indicate the groups that are statistically indistinguishable from one another (ANOVA and pairwise t tests, p < 0.05). The supplementary file in which the data used to generate each plot can be found is S1 Data. (TIF) [file pbio.1002392.s006.tif]

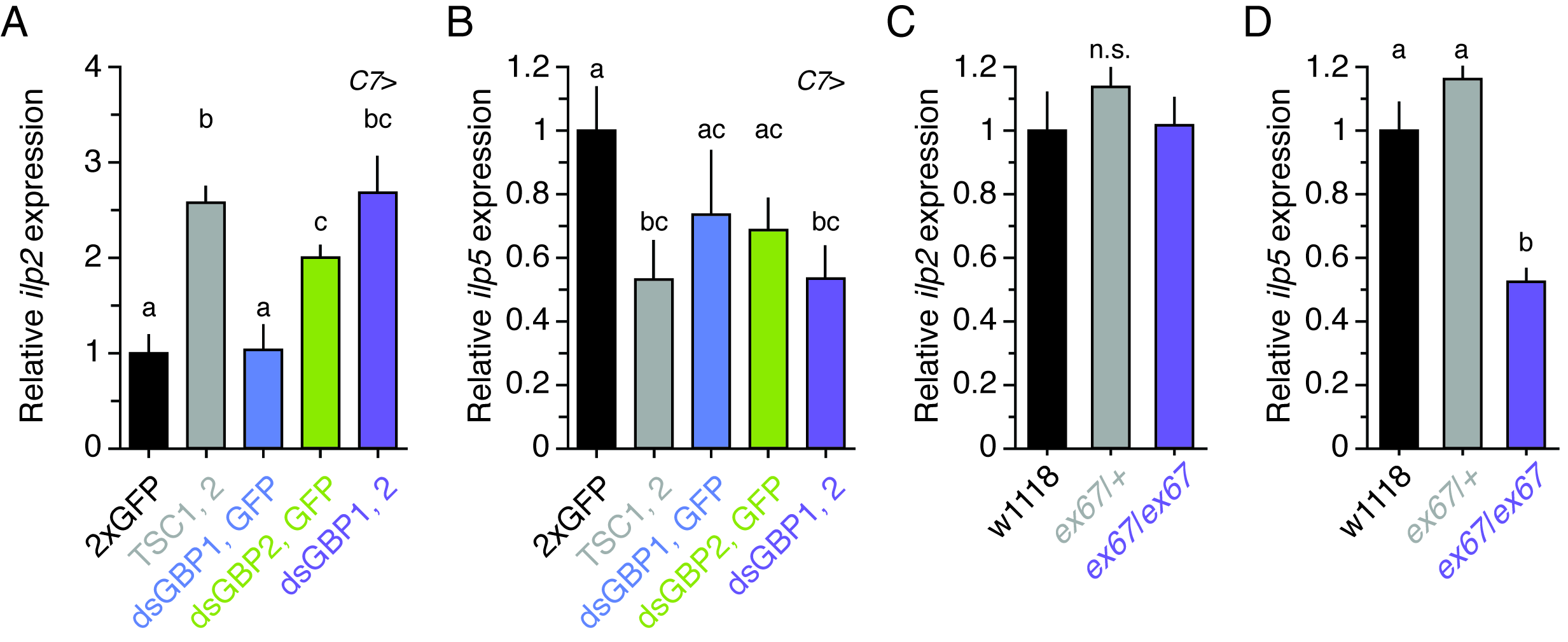

Supplement: S6 Fig — (A, B) GBP1, GBP2 double-knockdown in the fat body increases ilp2 expression (A) but reduces ilp5 expression (B). To control for transgene copy number, we overexpressed two copies of UAS transgenes using the C7 Gal4 driver. (C, D) The gbp1, gbp2 ex67 mutant shows no effect on ilp2 expression (C) but decreases ilp5 expression (D). We normalized the values using an internal control, RpL3. Then, we standardized the expression level of each gene by fixing the values from C7>2xGFP or w1118 animals to 1. We used five larvae for each sample and five biologically independent samples for each genotype. Each bar indicates the relative mean expression ± SEM. Treatments sharing the same letter indicate the groups that are statistically indistinguishable from one another (ANOVA and pairwise t tests, p < 0.05). The supplementary file in which the data used to generate each plot can be found is S1 Data. (TIF) [file pbio.1002392.s007.tif]

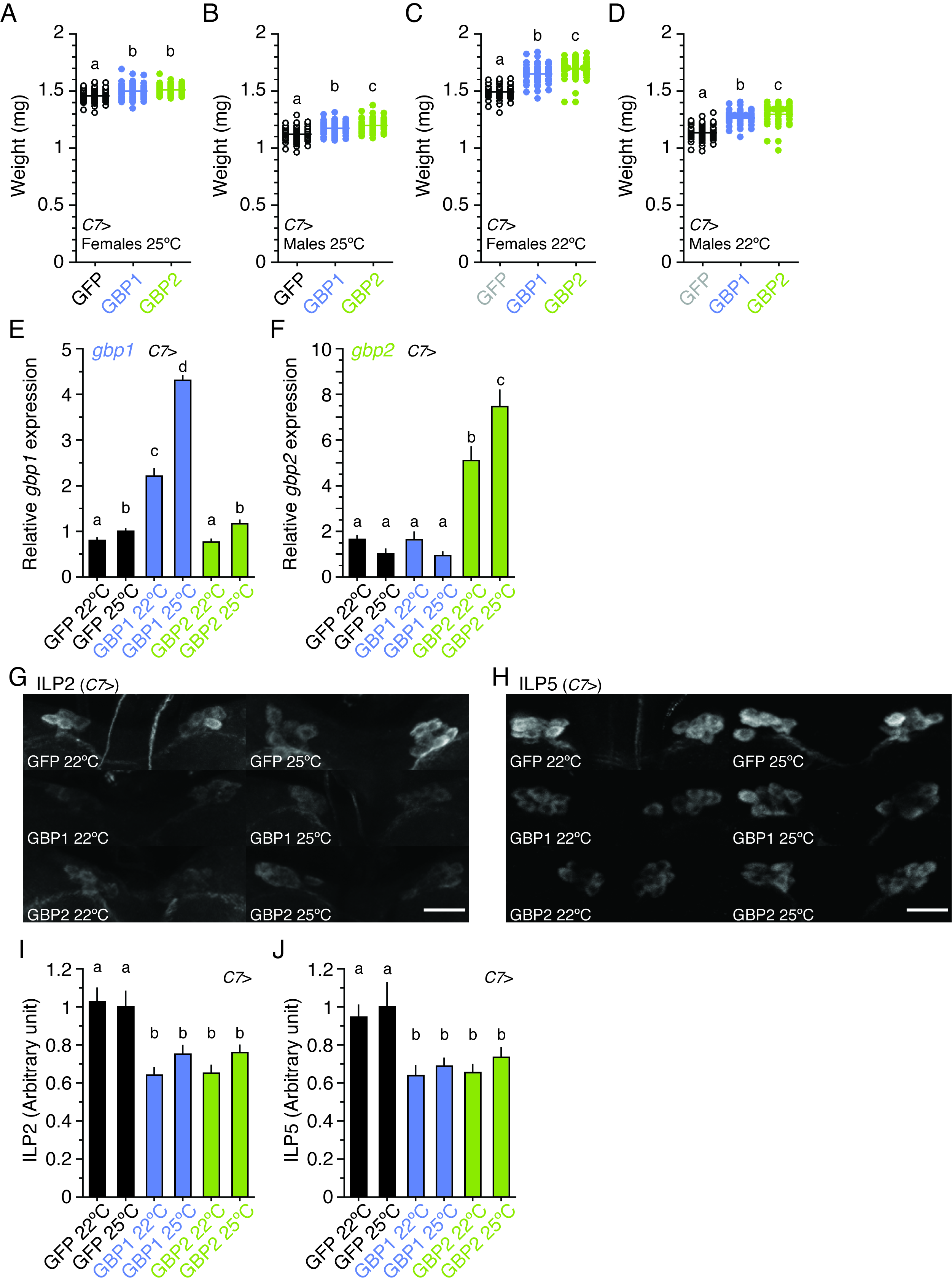

Supplement: S7 Fig — (A-D) Overexpressing GBP1 or GBP2 increases body size in wild-type females and males (A and B, respectively) at 25°C or 22°C (C and D, respectively). One copy of UAS transgenes was expressed using the C7 Gal4 driver. n = 70 for A, 69–72 for B, 61–74 for C, and 59–68 for D. (E, F) Expression level of GBP1 (E) or GBP2 (F) at 25°C is higher than that of 22°C. We normalized the values using an internal control, RpL3. Then, we standardized the expression level of each gene by fixing the values from C7>GFP at 25°C to 1. We used five larvae for each sample and five biologically independent samples for each genotype. Each bar indicates the relative mean expression ± SEM. (G, H) Overexpressing GBP1 or GBP2 at two different temperatures shows reduced ILP2 (G) and ILP5 (H) accumulation in the insulin-producing cells. The scale bars are 20 μm. (I, J) Overexpressing GBP1 or GBP2 at two different temperatures shows reduced densities of ILP2 (I) and ILP5 (J) signals in the insulin-producing cells. We standardized the densities of ILPs by fixing the values from C7>GFP at 25°C to 1. n = 19–46 for both ILP2 and ILP5. Treatments sharing the same letter indicate the groups that are statistically indistinguishable from one another (ANOVA and pairwise t tests, p < 0.05). The supplementary file in which the data used to generate each plot can be found is S1 Data. (TIF) [file pbio.1002392.s008.tif]
