# Supplementary material for: Growth-Blocking Peptides As Nutrition-Sensitive Signals for Insulin Secretion and Body Size Regulation
Source: PLoS Biol. 2016 Feb 29;14(2):e1002392. doi: 10.1371/journal.pbio.1002392 (PMC4771208; doi:10.1371/journal.pbio.1002392)
Supplement: S1 Table — (DOCX) [file pbio.1002392.s009.docx]

| Gene | Purpose | Sequence (5’-3’) | Ref |
| --- | --- | --- | --- |
| *gbp1* | UAS construct | F: 5’-CACCAGATCTCGAAGCTTCATCGTAGCAAAC-3’  R: 5’-TCTAGATTACGCCGGCTTTCTGCATC-3’ | This work |
| *gbp2* | UAS construct | F: 5’-CACCAGATCTTCAGTCTGTTTTGAATATTCAACGCTGCC-3’  R: 5’-TCTAGACTAGGCTTCCTTCCTGCAAC-3’ | This work |
| *gbp1* | qPCR | F: 5’-ATCCTACCGCTGGTCTTCCTC-3’  R: 5’-CTCCAGCAATATTCGGTTGTC-3’ | This work |
| *gbp2* | qPCR | F: 5’-CGCCTCCTTCGTATTATCCAG-3’  R: 5’-CCAGATGGTTGTGGTCTATTG-3’ | This work |
| *gbp3* | qPCR | F: 5’-TGGGCAGAATTTTCCACAGGG-3’  R: 5’-GTCTTCTCGGTGGTGGTTTGG-3’ | This work |
| *gbp2* | qPCR for *ex67* mutation | F: 5’-AGAGTACTGGGGGATTCCAACAG-3’  R: 5’-TCTTCATCTGCGCTTCCGGGAC-3’ | This work |
| *ilp2* | qPCR for *ex67* mutation | F: 5’-ACGAGGTGCTGAGTATGGTGTGCG-3’  R: 5’-CACTTCGCAGCGGTTCCGATATCG-3’ | [44] |
| *ilp5* | qPCR for *ex67* mutation | F: 5’-TGTTCGCCAAACGAGGCACCTTGG-3’  R: 5’-CACGATTTGCGGCAACAGGAGTCG-3’ | [44] |
| *gal4* | qPCR | F: 5’-CCGACCATGATTACGGATAGATAC-3’  R: 5’-AACCTGACTCGAAGACCTTGCTCG-3’ | This work |
| *RpL3* | qPCR | F: 5’-AAGGATGACGCCAGCAAGCCAGTC-3’  R: 5’-TAGCCGACAGCACCGACCACAATC-3’ | [27] |
